# Exploring interactions of *Aliivibrio fischeri* with water-soluble polymers using bioluminescence and Raman microspectroscopy

**DOI:** 10.1371/journal.pone.0330775

**Published:** 2025-09-16

**Authors:** Thomas J. Tewes, Britta Brands, Felix H. Schacher, Dirk P. Bockmühl

**Affiliations:** 1 Faculty of Life Sciences, Rhine-Waal University of Applied Sciences, Marie-Curie-Straße 1, Kleve, Germany; 2 Dr. Brill + Prof. Bockmühl GmbH, Wiesenstraße 35, Kleve, Germany; 3 Institute of Organic Chemistry and Macromolecular Chemistry (IOMC), Friedrich-Schiller University Jena, Humboldtstraße 10, Jena, Germany; 4 Jena Center for Soft Matter (JCSM), Philosophenweg 7a, Jena, Germany; Maulana Abul Kalam Azad University of Technology West Bengal, INDIA

## Abstract

Water-soluble polymers (WSPs) are widely used in biomedical and industrial applications. However, their ecological impact, including interactions with microorganisms, remains insufficiently understood and warrants further investigation. *Aliivibrio fischeri*, a bioluminescent bacterium, serves as a sensitive model to explore these effects. This study takes an exploratory approach, combining the optical spectroscopic techniques of luminescence measurements and Raman microspectroscopy to assess both immediate metabolic responses and potential longer-term or structural changes. Four WSPs polyacrylamide (PAM), polyethylene glycol (PEG), polyvinyl alcohol (PVOH), and polyvinylpyrrolidone (PVP) were each tested with three different molecular weights and five concentrations for luminescence measurements based on DIN EN ISO 11348. The tests revealed polymer-specific effects: PAM, PEG, and PVP suppressed luminescence, likely due to osmotic stress, adsorption, or viscosity-related limitations, with more pronounced inhibition observed at higher molecular weights. The strongest luminescence reduction was observed for PEG. Unlike the toxic reference substance 3,5-dichlorophenol (DCP), polymer-induced luminescence changes did not follow a consistent monotonic decay, suggesting that their effects are not solely attributable to acute toxicity. Notably, PVOH exposure increased luminescence, potentially reflecting stabilizing interactions or improved oxygen availability, which clearly contrasts with the suppressive trends observed for the other polymers. To investigate potential biochemical alterations, we applied Raman microspectroscopy to polymer-exposed *A. fischeri*. Partial least-squares discriminant analysis (PLS-DA) identified spectral differences, with polymer-specific patterns observed in regions commonly associated with membrane components, DNA, or carbohydrates. However, the PLS-DA coefficients primarily reflect statistical relevance and do not directly indicate specific biochemical mechanisms. These findings highlight spectral signatures relevant for classification, while biological interpretation requires further investigation. Our study provides exploratory insights into the potential impact of WSP exposure on bacterial metabolism and cellular composition, demonstrating the value of combining luminescence and Raman spectroscopy to detect polymer-related effects on microorganisms. While the observed spectral and metabolic changes suggest polymer-specific interactions, further research is needed to confirm the underlying mechanisms and assess their broader environmental and biotechnological implications.

## Introduction

Water-soluble polymers (WSPs) are widely utilized in pharmaceuticals [1–3], agriculture [4,5], serving as thickeners [1,6], dispersants [7], and stabilizers [8–10]. Due to their hydrophilic nature and functional versatility, WSPs play a role in numerous formulations, but concerns regarding biodegradability, bioavailability, and environmental persistence have raised questions about their potential effects on microbial systems [11]. Among WSPs, polyacrylamide (PAM), polyethylene glycol (PEG), polyvinyl alcohol (PVOH), and polyvinylpyrrolidone (PVP) are particularly relevant due to their diverse applications and varying physicochemical properties [12]. Each of these polymers exhibits distinct chemical characteristics that may influence bacterial systems differently. PAM is widely used in wastewater treatment and soil conditioning due to its flocculant properties [13], yet concerns persist regarding its environmental impact [14,15]. PEG plays a crucial role in pharmaceutical and cosmetic formulations, improving solubility and controlled drug release [16], but its long-term environmental impact remains debated [11]. PVOH, valued for its film-forming and adhesive properties [17], is often incorporated in soluble detergents packaging [18], yet issues regarding biodegradability have emerged [19]. PVP serves as a binder and solubilizer in various formulations [9,20,21], though data on its long-term ecological effects remain limited [22–25]. Due to their solubility in water, these polymers may show even more versatile interactions with microorganisms than solid, insoluble particles. The solubility of polymers in water depends on structural properties. For example, a higher number of heteroatoms such as nitrogen and oxygen increases the polarity, which improves water solubility [26]. WSPs contain hydrophilic groups, such as hydroxyl (-OH) in PEG and PVOH, and amide groups in PAM and PVP, which form hydrogen bonds with water [27–30]. Flexible polymer chains further facilitate dissolution [31,32], as they enable effective hydration by surrounding water molecules [33,34], promoting polymer-water interactions [35]. Low intermolecular interactions prevent strong intra- and interchain bonds, improving solubility [36]; for instance, PEG’s linear structure and weak intermolecular forces aid dissolution. Low steric hindrances can also enhance solubility, as seen in PVP, where bulky pyrrolidone side groups prevent tight chain packing [37]. While ionic groups like carboxylate or ammonium increase solubility through electrostatic interactions [38], they are not relevant to the polymers studied here. The use and environmental release of WSPs increases and understanding their potential interactions with microorganisms becomes increasingly relevant, particularly in connection with ecological risk assessment and biocompatibility [39]. In this context, *Aliivibrio fischeri*, a marine bacterium known for its bioluminescence, can serve as a sensitive model organism for investigating biological responses to chemical stimuli [40]. Yet, the mechanisms by which WSPs influence microbial physiology at the molecular level remain poorly understood [41,39]. Bacteria play a key role in environmental polymer degradation, but WSPs may also influence bacterial physiology, metabolism, or surface properties. Understanding these interactions is essential for assessing potential microbial responses to polymer exposure in both natural ecosystems and industrial applications. *A. fischeri* is a well-established model organism for studying environmental stressors due to its natural bioluminescence, which is tightly coupled to cellular metabolism [42]. The light emission results from the enzymatic activity of luciferase, encoded by the luxA and luxB genes, which catalyzes the oxidation of a long-chain aldehyde (e.g., decanal) and the reduction of FMNH_2_ [43–45]. This process emits light at approximately 495 nm. The remaining genes of the lux operon are involved in substrate recycling and regulation [46,47]. Bioluminescence intensity is regulated via quorum sensing, involving the LuxR–LuxI system and the synthesis of N-acyl homoserine lactones [48–50]. Light output is highest under optimal pH (7.8) and temperatures (20–30°C), and even low concentrations of 120 nM of homoserine lactone can induce maximal luminescence [48,49]. This mechanism is applied in DIN EN ISO 11348, which uses *A. fischeri* luminescence as a sensitive indicator for toxic effects in water samples.

In this study, we made use of the described luminescence mechanism of *A. fischeri* and examined the WSPs PAM, PEG, PVOH, and PVP with three different molecular weights, five concentrations and different contact times to explore potential interactions. Instead of measuring luminescence at a single wavelength, we recorded entire luminescence spectra for a more comprehensive assessment. Optical spectroscopic methods in general provide powerful tools for analyzing molecular interactions, offering insights into chemical composition, structural characteristics, and physicochemical properties across a wide range of applications [51–54]. These methods, including Raman spectroscopy, have gained attention in polymer characterization due to their non-destructive nature and their capability to provide molecular-level insights through vibrational analysis [12]. However, while optical spectroscopic approaches have been used in polymer chemistry and material science, their potential in assessing polymer-microorganism interactions remains underexplored. In addition to the luminescence tests, we collected extensive Raman microscopic data to develop classification models using PLS-DA, training three models to differentiate between polymer exposure in general, individual polymer types, and specific molecular weights. By analyzing PLS-DA coefficients, spectral regions that are particularly relevant for classification could be identified and further examined. By combining functional metabolic data with molecular-level spectral analysis, this study takes an exploratory approach to assess whether Raman spectroscopy can detect bacterial responses to polymer exposure and to identify polymer-specific effects. Given the increasing environmental and industrial relevance of WSPs, these findings contribute to a broader understanding of bacteria-polymer interactions and highlight the need for further studies to elucidate underlying mechanisms.

## Materials and methods

### Preparation of WSP solutions

**Fig 1:**
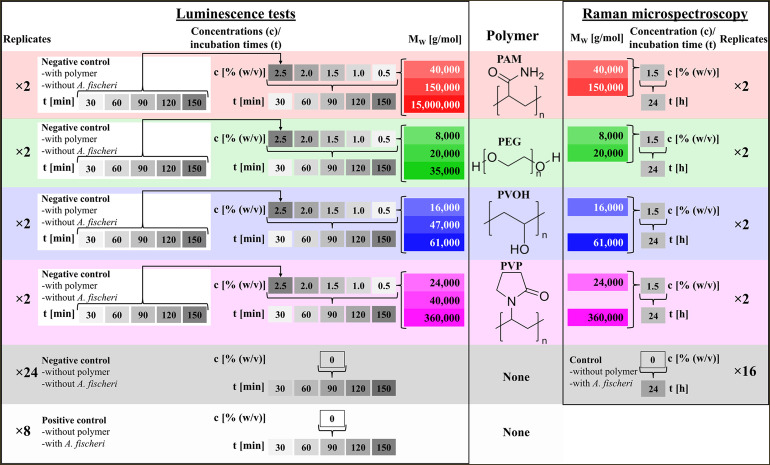
Chemical structural formulas of the investigated polymers and general overview of the analyzed sample preparations for luminescence tests and Raman microscopic analysis, considering molecular weights, concentrations, incubation times, and the types of controls used. For luminescence assays, a total of 8 positive controls were included to ensure comparability across experimental plates. Each polymer group (three molecular weights) was tested on a shared 96-well plate, with duplicate positive controls using the same *A. fischeri* culture included per plate.

The polymers used in this study were PAM, PEG, PVOH and PVP (Fig 1). For the luminescence assays, stock solutions with a concentration of 2.5% (w/v) were prepared by dissolving 2.5 g of each polymer, weighed on an analytical balance (XA105, Mettler-Toledo, Gießen, Germany), in sterile ultrapure water (Direct-Q 3UV Water Purification System, Merck KGaA, Darmstadt, Germany) under stirring at room temperature. After complete dissolution, the solutions were filled up to a final volume of 100 mL. From each stock solution, four dilutions were prepared to yield final polymer concentrations of 2.5%, 2.0%, 1.5%, 1.0%, and 0.5% (w/v) (corresponding to 25,000 mg/L down to 5,000 mg/L, equivalent to 25,000,000–5,000,000 µg/L). The tested concentration range was selected to span a broad gradient of exposure levels. This enabled the detection of both subtle and more pronounced physiological responses of *A. fischeri* to polymer treatment and allowed for potential concentration-dependent effects to be evaluated. In contrast to previous studies that reported no clear luminescence effects at lower concentrations [55], the present study deliberately applied higher concentrations to systematically explore possible threshold behaviors and ensure observable responses even at elevated exposure. Due to its lower solubility, PVOH was dissolved at approximately 70°C. Additionally, a highly soluble PVOH control solution was prepared to exclude potential effects caused by the modified dissolution procedure (S1 File). For Raman spectroscopy, separate polymer solutions were prepared at an initial concentration of 7.5% (m/v) by dissolving the respective polymers directly in 20 g/L NaCl solution, following section 5.2 DIN EN ISO 11348–1:2009-05 [56]. To investigate the influence of polymer exposure on the luminescence of *A. fischeri*, three molecular weight variants were tested for each polymer (Table 1). The tested molecular weights were selected to represent a broad range of polymer chain lengths, while ensuring comparability across different WSP types. In contrast to previous studies using short-chain WSPs [55], the present study included higher molecular weights (≥8000 g/mol) to investigate potential size-dependent effects on *A. fischeri* bioluminescence. The tested concentration range was selected to span several orders of magnitude, enabling assessment of both potential thresholds for physiological responses and general trends in concentration-dependent effects. It also facilitates comparison with previous laboratory studies on microbial sensitivity to polymer exposure.

**Table 1:** WSPs, their used abbreviations, M_W_ according to supplier specifications, and experimentally determined M_W_, M_n_, and Ð measured by SEC [12], along with supplier information.

Polymer	Abbreviation	M_W_ (Supplier) [g/mol]	M_W_ (SEC) [g/mol]	M_n_ (SEC) [g/mol]	Đ (SEC)	Supplier
Polyacrylamide	PAM	40,000	204,700	91,100	2.25	Sigma-Aldrich Chemie GmbH, Taufkirchen, Germany
		150,000	325,600	151,100	2.15	
		15,000,000	1,824,000	195,100	9.35	Carl Roth GmbH & Co. KG, Karlsruhe, Germany
Polyethylene glycol	PEG	8,000	8,300	7,700	1.08	Carl Roth GmbH & Co. KG, Karlsruhe, Germany
		20,000	22,900	19,900	1.15	
		35,000	38,100	31,100	1.23	Sigma-Aldrich Chemie GmbH, Taufkirchen, Germany
Polyvinyl alcohol	PVOH	16,000	57,100	32,200	1.77	Carl Roth GmbH & Co. KG, Karlsruhe, Germany
		47,000	103,500	59,900	1.73	
		61,000	304,000	185,300	1.64	
Polyvinylpyrrolidone	PVP	24,000	39,400	14,700	2.68	Carl Roth GmbH & Co. KG, Karlsruhe, Germany
		40,000	57,800	15,400	3.75	
		360,000	728,000	382,000	1.91	Sigma-Aldrich Chemie GmbH, Taufkirchen, Germany

The WSPs used in this study, along with their abbreviations, suppliers, and manufacturer-reported molecular weights, are summarized in Table 1. The molecular weights (M_W_), number-average molar masses (M_n_) and polydispersity (Ð) of all polymers investigated in this study were analyzed using size exclusion chromatography (SEC). The methodology for this and additional Raman spectroscopic analysis of the same raw material can be found in Tewes *et al.* [12].

### Luminescence measurements

#### Bacterial stock suspensions.

A lyophilized culture of *A. fischeri* (DSM-No. 7151) was purchased from German Collection of Microorganisms and Cell Cultures GmbH (DSMZ). The lyophilized cells were rehydrated in liquid medium according 5.7 DIN 11348 [56] and then grown on agar plates according 5.8 DIN 11348 [56] at 20°C for 48 hours. Luminescent single colonies were identified and marked in the absence of light. The Petri dishes were then stored in a refrigerator at 4°C for two weeks. The identified colonies were transferred to fresh Petri dishes under sterile conditions and incubated at 20°C for further 48 hours. From this culture, a 2-day-old single light-emitting colony was transferred to 50 ml of preculture medium (5.7 DIN 11348 [56]) and shaken for 21 hours at 20°C at 180 min^−1^ (KS 4000 i control, IKA-Werke GmbH & Co. KG, Staufen, Germany). The remaining steps up to the preparation of the stock cultures correspond to chapters 8.2 to 8.4 of DIN 11348 [56]. The stock suspensions aliquoted in 100 µl were then stored in a freezer at −80°C until use.

#### Sample preparation.

All prepared polymer solutions were analyzed for their pH value (Seven Compact pH/Ion S220, Mettler-Toledo, Gießen, Germany) and the dissolved oxygen of the highest concentrated solution was measured (VOLTCRAFT DO-101, Conrad Electronic SE, Hirschau, Germany). For the luminescence tests, the samples must have a 20 g/l NaCl concentration according to the standard [56], which is why salt was weighed into each polymer test solution accordingly. Particularly with more viscous samples, dissolving the salt may require prolonged shaking. In white 96 well microtiter plates with U-bottom (BRAND plates, VWR International GmbH, Darmstadt, Germany), 150 µl of that polymer solutions were added as duplicates. In addition, the control solutions 3,5-dichlorophenol (DCP) (Sigma-Aldrich Chemie GmbH, Taufkirchen, Germany) and pure salt solution with 20 g/l according 5.2 DIN 11348 [56] were also pipetted as duplicates. DCP, which is a well-known toxic substance for *A. fischeri*, was prepared at a concentration of 9 mg/L according to DIN 11348 [56]. 150 µl saline solution with 150 µl bacterial suspension was used as the positive control, and 300 µL of saline solution without bacteria was used as the negative control. Additional negative controls without *A. fischeri* but with the highest polymer concentration were also performed by mixing 150 µl NaCl solution with 150 µl polymer solution. The logic and terminology of the test preparations can be seen in Fig 1. To ensure comparability across all luminescence measurements, each experimental setup involving a specific polymer was accompanied by a dedicated positive control using the exact same *A. fischeri* culture. Since all three molecular weight variants of one polymer were analyzed together on a single 96-well plate, two replicate wells containing the unexposed culture were included on each plate. As four such polymer-specific plates were used, a total of eight replicate positive controls were conducted (Fig 1).

The aliquots with the luminescent bacteria were thawed at 20°C and mixed with 0.5 ml solution for freshly cultivated bacteria (5.5 DIN 11348 [56]) precooled to 15°C. The suspension was then incubated at 15°C for 15 minutes (ThermoMixer C, Eppendorf Vertrieb Deutschland GmbH, Wesseling, Germany). That solution was transferred to 2.25 ml solution for freshly cultivated bacteria (5.5 DIN 11348 [56]) at 15°C and incubated at 15°C for a further 15 minutes. Depending on how many wells of a microtiter plate were filled, several aliquots of the stock suspensions were used, which were initially incubated individually with 0.5 ml solution for freshly cultivated bacteria and then transferred together to the correspondingly larger volume of that medium. A timer was started and 150 µl of the incubated bacterial suspension was added to the first sample while gently stirring with the pipette tip to achieve proper mixing. Before the bacterial solution was added to the next sample, 30 seconds elapsed. Each sample was therefore pipetted with an interval of 30 seconds to the previous sample. The total volume of each well chamber was 300 µl.

#### Measurement of luminescence spectra.

Complete luminescence spectra in the wavelength range from 460 to 530 nm were recorded using a spectral scanning multimode reader (Varioskan Flash, Thermo Fisher Scientific GmbH, Dreieich, Germany). The intensity of the luminescence was measured every 2 nm, so that a luminescence spectrum consists of 36 measuring points. The measurement time of each wavelength was 850 ms, which means that the measurement of an entire luminescence spectrum took about 30 seconds. The measuring time of a sample therefore corresponds to the contact time of the bacteria with the sample material. The samples were measured in the order of the previous pipetting after a total contact time of 30 minutes, then every 30 minutes up to and including 150 minutes (30 min, 60 min, 90 min, 120 min, 150 min). Thus, 5 concentrations and 5 incubation times were examined for each polymer test preparation (see Fig 1). By comparing the decrease in luminescence of a sample with the decrease in luminescence of a corresponding positive control, statements can be made about the influence of the test substances on the luminescence of *A. fischeri*.

### Raman microspectroscopy

#### Bacterial growth and polymer exposure.

For the Raman microspectroscopic approach the same *A. fischeri* strain was used as in the luminescence measurements, but separate cultures were used. As in the luminescence tests, lyophilized cells were rehydrated in liquid medium [56] and subsequently grown on agar plates according to section 5.8 DIN 11348 [56] at 20°C for 48 hours. Luminescent single colonies were transferred to fresh agar plates under sterile conditions and incubated at 20°C for 48 hours at 50% humidity (HP110, Memmert GmbH & Co. KG, Schwabach, Germany). The cell material was flushed from the agar plates with sterile spatulas using 10 ml of 20 g/L NaCl solution. The bacterial suspension was mixed with 10 ml sterile 80% glycerol (VWR International GmbH, Darmstadt, Germany) and 500 µl aliquots were prepared from this, which were then frozen and stored at −80°C. These stocks were used as initial cultures by spreading the entire volume on agar plates and incubating the cultures at 20°C for 72 hours at 50% humidity.

As much material of a 72 hours grown initial culture was transferred into 20 mL medium according 5.7 DIN 11348 [56] in a 50 mL centrifuge tube (Sarstedt AG & Co. KG, Numbrecht, Germany) using an inoculation loop until an OD_600_ value between 0.520 to 0.580 was reached. The samples were homogenized on a vortex mixer and then shaken and incubated at 750 rpm (ThermoMixer C, Eppendorf Vertrieb Deutschland GmbH, Wesseling, Germany) at 20°C for 24 hours. These liquid cultures are called starting cultures in our study. Following Tewes *et al.* [12], two independent batches were prepared for each test polymer and control (sample preparations without polymer exposure). For polymer exposure, 7.5% (w/v) solutions of the polymers shown in Table 2 were prepared following the same procedure as described above for the luminescence experiments, but using 20 g/L NaCl (according 5.2 DIN 11348 [56]) as solvent (corresponding to 75,000 mg/L or 75,000,000 µg/L) Three mL of medium according 5.7 DIN 11348 [56] was mixed with 1 mL of 7.5% (m/v) polymer solution and 1 mL of the 24 h liquid culture (starting culture) in 15 mL centrifuge tubes, resulting in a polymer concentration of 1.5% (m/v). As controls without any polymer, three mL of medium according 5.7 DIN 11348 [56] was mixed with 1 mL of the 20 g/L NaCl solution and 1 mL of the 24 h incubated starting culture. These test and control preparations were incubated for a further 24 hours. The lids of the centrifuge tubes were always loosened during incubation. OD_600_ values of all starting cultures, test and control preparations were determined before and after incubation (Biophotometer plus, Eppendorf SE, Hamburg, Germany) to ensure standardized conditions and to monitor any differences that might influence the experiments. For all OD_600_ measurements, 800 μl volumes of corresponding solutions were used. Results of OD_600_ monitoring can be found in S2 File.

**Table 2:** Overview of systematic sampling for Raman microscopic analysis.

Polymer	Molecular weights [g/mol]	Individual batches	Measurement areas per batch	Spectra per area	Spectra overall
PAM	40,000	2	4	30	240
	150,000	2	4	30	240
PEG	8,000	2	4	30	240
	20,000	2	4	30	240
PVOH	16,000	2	4	30	240
	61,000	2	4	30	240
PVP	24,000	2	4	30	240
	360,000	2	4	30	240
Controls		16	4	30	1920
Sum					3840

#### Sample purification and preparation.

After final incubation of the test and control preparations, purification was carried out to get rid of polymer residues. This process was described in a similar way in Tewes *et al.* [12]. To treat both test and control preparations identically, the purification procedure was also carried out in parallel for the controls. The samples were centrifuged at 4696 × g at 20°C for 10 minutes. The supernatants were discarded and any residual liquid on the cell pellets were carefully aspirated using a piston-stroke pipette. Then the cell pellets were homogenized in 2 mL of 20 g/L saline solution each on the vortex mixer and centrifuged again under the same conditions. Again, the supernatants were removed completely and this time the cell pellets were carefully resuspended in 2 mL of 20 g/L NaCl solution and the whole volumes were transferred to new sterile 15 mL centrifugation tubes, mixed on the vortex mixer and centrifuged again. This was followed by a third and final washing step as described above, including complete removal of the supernatant. The final cell pellets were then resuspended in 125 µl 20 g/L NaCl and one µl droplets were pipetted onto a clean sterile polished stainless-steel slide (Renishaw, Pliezhausen, Germany). When pipetting the droplets on the steel slide, care was taken to ensure that the drops of all samples were applied in the same way, i.e., that the drop shape itself did not vary greatly. The stainless-steel slide was transferred to an incubator in an empty sterile Petri dish with an angled lid and the droplets dried at 20°C and 50% humidity for 40 minutes. The actual drying takes place after about 15 minutes, but the additional time was added to ensure that all residual moisture was removed, and uniform measuring conditions were guaranteed.

#### Spectral recording.

The confocal Raman system used (inVia, Renishaw, Gloucestershire, UK) includes a Helium-Neon (HeNe) laser with an excitation wavelength of 633 nm. The selected spectral region was set to 606–1736 cm ⁻ ¹, and the spectral resolution was about 1.1 cm ⁻ ¹ (1800 l/mm grating). For focusing samples, a 100 × magnification lens (numerical aperture of 0.85) was used. The laser power was set to 50%, which means approximately 3.5 mW on sample. The laser diameter with the setup used is about 7–8 µm. Four individual areas were examined on the sample, where 30 spectra were recorded in a spiral arrangement from the center to the outside with a distance of about 2–4 μm between the measuring spots, similar to Tewes *et al.* [57]. Exposure time was 1.5 seconds and 20 accumulations per spectrum were acquired. The default cosmic ray removal filter of the Wire 4.4 Raman system software (Renishaw, Gloucestershire, UK) was activated during spectral acquisition. The four measurement areas of dried sample droplets were first localized with a 20 × lens and then sampled with the 100 × lens. Two of the four locations of an individual sample were close to the center of each droplet, one in the middle edge region of the droplets and one at the outermost edge. Since the salt concentration was very high (20 g/L) and the droplets partially crystallized, care was taken to examine areas that did not show any visible salt crystallization effects. S3 File shows exemplary microscopic images to illustrate crystallized areas and the selection of measurement areas for analysis.

In order to exclude any time-related influence on Raman spectra during the measurement procedure, each spiral of 30 spectra was always measured alternately from the test preparation and then from the control preparation. Two selected molecular weights were examined for each of the four polymers (PAM, PEG, PVOH, PVP), two independent batches (see Fig 1), each with four different measurement areas and 30 individual measurements were carried out, resulting in 240 Raman spectra being recorded for each individual test batch. Since controls without polymer exposure were always included, there are also 240 control spectra for each of the 8 test approaches. This brings the number of Raman spectra with polymer exposure to 1920 and the number of control spectra to 1920, giving a total of 3840 Raman spectra. Table 2 shows this breakdown in detail. The chosen molecular weights are based on the results of the luminescent bacteria tests and explained in the results section.

### Data pre-processing and analysis

#### Luminescence spectra.

The luminescence spectra were not pretreated, but the intensities of each wavelength of a spectrum were summed to determine the light intensity. The individual sums of both spectra of a double determination were arithmetically averaged. This arithmetic mean of the summed intensities was presented as a percentage in relation to the corresponding positive control whose light intensity was calculated in the same way. A sample luminescence of 100% therefore means that the sample preparation has the same light intensity as the positive control at the same contact/incubation time.

#### Raman spectroscopic data.

The spectra were processed according to Tewes *et al.* [57] where MATLAB R2022b (MathWorks, Massachusetts, USA) was used to read in the Raman spectra, interpolate and implement the baseline correction using the “msbackadj” function with window size 50 and step size 50. Smoothing was performed via Savitzky-Golay filtering (polynomial order 3, window size 13). The spectra were z-score normalized before the data were used for modeling.

The classification toolbox for MATLAB version 7.0 from Ballabio and Consonni [58] was used to train venetian blind five-fold cross-validated PLS-DA models. Since spectra were already pre-treated the setup for row pre-processing was set to “none”, column pre-processing was set to “autoscaling” and assignation criterion to “max”. Overall, three individual PLS-DA models with increasing complexity were calculated after determining the optimal number of latent variables (LVs).

**Two-class model:** PLS-DA using 4 LVs to differentiate Raman spectra of *A. fischeri* exposed to polymers from Raman spectra of controls, i.e., *A. fischeri* without any contact to polymers.

**Five-class model:** PLS-DA using 14 LVs to differentiate Raman spectra of *A. fischeri* exposed to the four individual polymer types PAM, PEG, PVOH, PVP and Raman spectra of controls.

**Nine-class model:** PLS-DA using 18 LVs to differentiate Raman spectra of *A. fischeri* exposed to the four individual polymer types PAM, PEG, PVOH, PVP in two different molecular weights each and Raman spectra of controls.

To evaluate class-wise model behavior, predicted response values from PLS-DA models were plotted for each class. These values result from projecting each spectrum onto the latent variables of the model’s class-specific component [58]. Higher positive values indicate stronger association with the respective class, while values near zero or negative suggest lower similarity. This visualization enables a qualitative assessment of class separability and potential spectral overlap and is complemented by performance metrics such as confusion matrices.

In addition, detailed confusion matrices of the five-fold cross-validated models were created which provide insights into classification performance by displaying the number of correctly and incorrectly predicted samples for each class, helping to identify potential misclassifications and model biases.

Finally, we use the PLS-DA coefficients to determine which regions within the Raman spectra contribute particularly to the classifiability of certain classes. By comparing the striking spectral regions with the corresponding biochemical origin of the signatures, first careful conclusions about possible interactions of *A. fischeri* with the polymers can be drawn.

## Results

### Luminescence measurements

**Fig 2:**
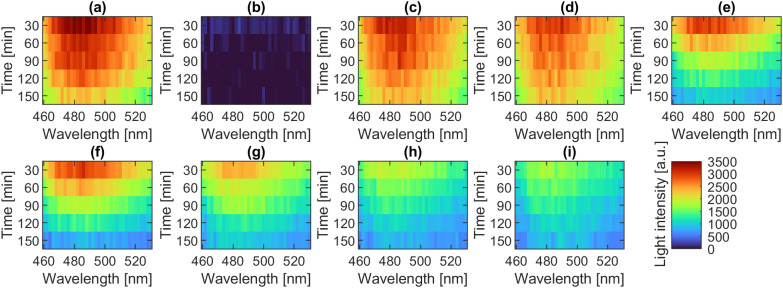
Luminescence spectra averaged from duplicate determinations shown as heat maps over the contact time. Positive control **(a)** and negative control **(b)**; exemplary the luminescence spectra of the lowest PVP concentrations (0.5%) of 24,000 g/mol **(c)**, 40,000 g/mol **(d)**, 360,000 g/mol **(e)**. As well as the remaining concentration levels of PVP at 360,000 g/mol namely 1% PVP **(f)**, 1.5% PVP **(g)**, 2.0% PVP **(h)**, 2.5% PVP **(i)**.

**Fig 3:**
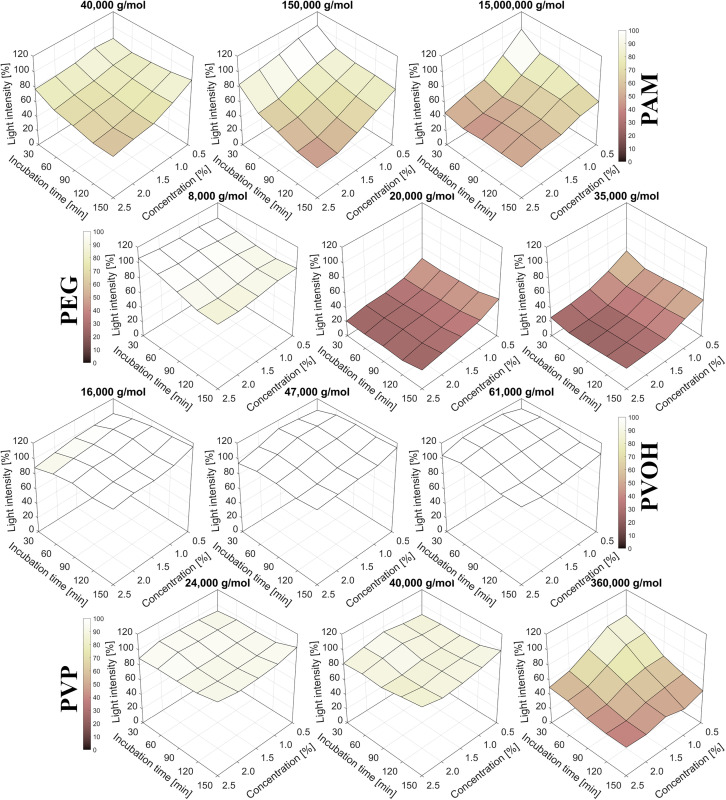
Luminescence intensity of *A. fischeri* after polymer exposure, shown as percentage relative to the corresponding positive control (100%). The surfaces illustrate the effect of polymer test concentration (% w/v) and incubation time. Note that the color scale is capped at 100% to emphasize reductions in signal; values exceeding 100% (as observed with some PVOH samples) appear as pure white.

The luminescence spectra recorded during the experiment illustrate the spectral behavior of *A. fischeri* under various exposure conditions. High emission intensities are visible in the range of 472–496 nm, particularly at early time points. In untreated bacterial suspensions (positive control), light emission is strongest at the beginning and decreases over time (Fig 2a), while no luminescence signal is detected in the negative control (Fig 2b), confirming its validity. Multiple types of negative controls were included, both with and without polymer exposure (Fig 1). None of these controls exhibited any measurable luminescence, as evidenced by the raw emission spectra provided in S4 File. As an example, the luminescence spectra of the PVP samples are shown in Fig 2c (24,000 g/mol), the relatively similar spectra at 40,000 g/mol in Fig 2d and the luminescence spectra of the 360,000 g/mol sample in Fig 2e, which are significantly weaker, especially after 90 minutes of contact time. The luminescence spectra of the higher PVP concentrations of 1% are shown in Fig 2f, 1.5% Fig 2g, 2% Fig 2h, 2.5% Fig 2i. Increasing polymer concentration results in a continuous suppression of luminescence. This concentration- and molar-mass-dependent pattern is clearly visible in the corresponding spectral heatmaps (Fig 2) and forms the basis for the subsequent integration and quantitative comparison in Fig 3. The characteristics described largely applies to the other polymers, but not consistently. Higher molar masses have no distinct effect on the luminescence of the PVOH samples and even increasing concentrations do not lead to a decrease in luminescence. In fact, the PVOH samples often emit more light than the positive control. This can be seen in Fig 3; there, the luminescence is shown relative to the corresponding positive control and all molar masses and concentrations of each tested polymer.

Another notable pattern in the PVOH luminescence data (Fig 3) is the initially reduced light emission after 30 minutes of incubation, followed by a gradual increase at later time points. This trend reflects a more rapid decline in luminescence in the corresponding positive controls. At 2.5% PVOH concentration, this leads to an initial luminescence of around 88%, which increases to around 96% after 60 minutes and reaches 101% after 90 minutes. After 120 minutes of incubation, the luminescence drops slightly to around 98% and rises again to 101% after 150 minutes. This temporal pattern is consistently observed across all tested PVOH molecular weights at 2.5% concentration, with only minor differences (Fig 3). A subtle tendency toward higher luminescence with increasing molecular weight is evident. The highest relative luminescence was observed for 61,000 g/mol PVOH at a concentration of 1.5% after 60 minutes incubation, reaching 116%.

The dissolved oxygen content was measured for the highest polymer concentration in each case and found to range between 7.6 and 8.8 mg/L, which is clearly above the DIN 11348 threshold of 3 mg/L (S5 File).

The pH values of the test solutions (polymer solutions) is ideally between 6.0 and 8.5 as extreme pH values can interfere with the test [56]. S6 File contains the measured pH values of all test solutions. Notably, the PVP solutions exhibit pH values below the recommended range. The upper limit value of 8.5 is only exceeded once with 2.5% PAM at 40,000 g/mol. The pH values of the polymer solutions were not adjusted beforehand due to the buffer capacity of the culture medium with which they were mixed during the test (see materials and methods). Thus, the 2.5% PVP sample (24,000 g/mol) measured with a pH of 3.82 was measured to be 6.67 after mixing with the culture medium. The lowest luminescence is present with PEG samples, except for the lowest molar mass tested (8,000 g/mol). However, the pH values do not show any extremes and in some cases are already within the ideal test range even without buffering; at the same time, the pH values of the PVOH samples before buffering (S6 File) were all slightly below 6 but show the highest measured luminescence.

Although PAM showed a faster decrease in light intensity with increasing molar mass (Fig 3), the initial intensity of the 150,000 g/mol sample at the lowest concentration (0.5%) and the shortest contact time (30 minutes) is comparable to the 15,000,000 g/mol sample (Fig 3). Furthermore, these two PAM samples with the higher molar masses differ significantly from each other (150,000 g/mol vs. 15,000,000 g/mol), whereas the luminescence was mainly noticeable at the higher concentrations (>1%) and short contact time (<90 min) (Fig 3).

**Fig 4:**
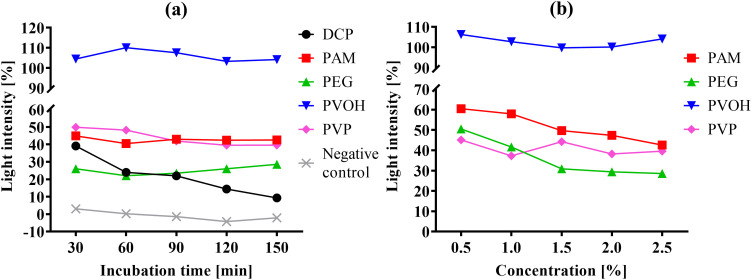
Relative luminescence (in % of positive control) over 150 minutes for the highest polymer concentration (2.5%), highest molar masses, and control samples including DCP and negative control (a). Luminescence profile as a dependence of the concentration of the polymer samples with the highest molar masses at the last measured time point (after 150 minutes incubation time) (b).

Fig 4a depicts 2-dimensional partial sections of the total overview in Fig 3 to illustrate the luminescence curves of the highest concentration (2.5%) of the highest molar masses of each polymer investigated. For comparison, the curve of the test substance DCP and a negative control (pure NaCl solution) is also shown. In this view (Fig 4a) it is particularly evident that the development of the luminescence of the polymer solutions differs from the development of the DCP sample. Although the 2.5% polymer samples emit a much weaker luminescence than the positive control (except for PVOH), there is no clear constant decrease as with DCP. In bacteria treated with PEG and PAM there is even a relative increase in luminescence, which does not mean that *A. fischeri* emits a stronger luminescence after a certain time than before, but rather that the decrease in luminescence per time (i.e., the gradient) is greater in the positive control than in the polymer sample; the absolute luminescence of all samples always decreases (as exemplary shown in Fig 2). Fig 4b illustrates the luminescence curves as a function of the concentration of the polymer samples with the highest molar masses at the last measured time point, i.e., after 150 minutes of incubation (b). Since only one concentration of DCP and only a single negative control condition were examined, they cannot be shown in Fig 4b. The PVOH preparation with 61,000 g/mol emits more light than the positive control after 150 minutes at almost all concentrations (Fig 4b); the luminescence falls from around 106% at the lowest concentration to just below 100% at the medium concentration of 1.5% and then rises again with increasing polymer concentration to about 104% at 2.5% polymer concentration. The curves for PAM and PEG show a systematic decrease in luminescence with increasing polymer concentration, whereas the corresponding curve for PVP differs in that the highest relative luminescence is at 1.5% polymer concentration (after 150 minutes) and there is no visible decrease with increasing concentration (Fig 4b).

Overall, PEG samples with molar masses of 20,000 g/mol and 35,000 g/mol show a distinct decrease in the luminescence of *A. fischeri* (Fig 3). No other WSPs show such a high loss of luminescence.

### Raman microspectroscopy

#### Raman spectra.

Of the three molecular weights tested for each WSP in the luminescence assays, two were selected for Raman microspectroscopic analysis: PAM 40,000 g/mol and 150,000 g/mol; PEG 8,000 g/mol and 20,000 g/mol; PVOH 16,000 g/mol and 61,000 g/mol; and PVP 24,000 g/mol and 360,000 g/mol. The selection aimed to capture the lowest and highest molar masses within each polymer group, thus reflecting the smallest and largest luminescence effects. However, practical and experimental constraints required slight adjustments: For PAM, the highest molecular weight (15 million g/mol) was excluded due to excessive viscosity at 7.5% w/v, which hindered handling. In the case of PEG, the 20,000 g/mol sample was chosen over 35,000 g/mol, as its effect on luminescence was more pronounced (see Fig 3).

**Fig 5:**
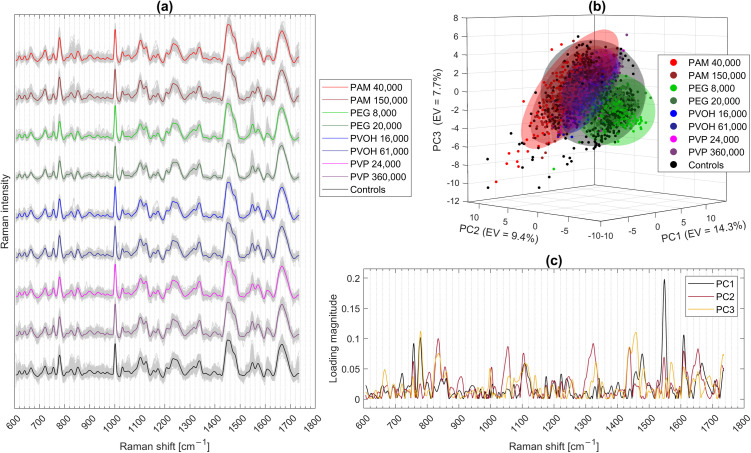
(a) Fully processed Raman spectra of *A. fischeri* incubated with the WSPs PAM, PEG, PVOH, and PVP in two different molecular weights each (values in g/mol given in the legend). Mean spectra are highlighted for each condition. Control spectra refer to bacteria without polymer exposure. The spectra were provided with a Y-axis offset for better visualization. (b) Principal Component Analysis (PCA) of the full Raman spectra using the first three PCs. A three-dimensional scores plot (PC1–PC3) is shown with convex confidence ellipsoids (95%) around each class with explained variance (EV) in %. (c) Contribution of each Raman shift to the first three principal components (PC1–PC3), expressed as the absolute loading magnitude.

The Raman spectra of all individual sample preparations are shown in gray, with mean spectra highlighted to emphasize central tendencies (Fig 5a). For each polymer type, two molecular weights and two independent batches were analyzed. From each dried sample, four independent measurement areas were selected, and 30 Raman spectra were acquired per area, resulting in a total of 480 spectra per polymer type (see Fig 1 and Table 2 for details). As controls without polymer contact were analyzed in parallel for each analysis of the samples with polymer incubation, there are (4x480) 1920 Raman spectra of the controls, also shown in Fig 5a.

Visually, the mean value spectra are almost identical (Fig 5a), suggesting only a minor influence of WSP exposure. In the Raman spectra of *A. fischeri* exposed to PEG, slightly elevated intensities around 1550 cm ⁻ ¹ are observed compared to the other WSPs, although similar variations are also present in control samples. This points to natural variability rather than a polymer-induced effect. Signals at approximately 826 and 853 cm ⁻ ¹ in PAM-exposed samples do not show major average changes but exhibit higher inter-sample variability, which occasionally exceeds that of the controls. Overall, Fig 5a illustrates the importance of chemometric approaches to uncover subtle class-dependent differences in complex Raman datasets. A Principal Component Analysis (PCA) was performed using all Raman spectra from the *A. fischeri* conditions shown in Fig 5a. The first three principal components (PC1-PC3) were retained for visualization. While no clearly separated clusters are apparent, the calculation of 95% confidence ellipsoids based on class-wise covariance matrices reveals initial patterns associated with polymer exposure (Fig 5b). To determine which spectral regions, contribute most to data discrimination, the loading vectors of PC1-PC3 were analyzed (Fig 5c). The absolute values of each loading vector were plotted to highlight the most influential Raman shifts, independent of their directional contribution in multivariate space.

#### Optimal number of LVs for PLS-DA models.

**Fig 6:**
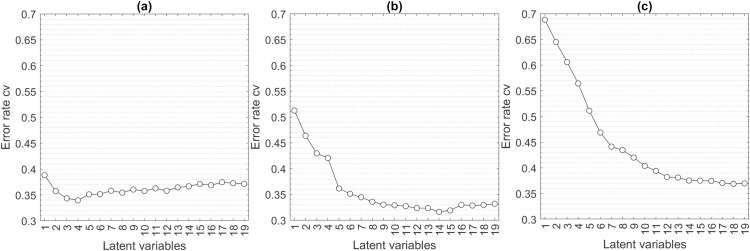
Five-fold cross-validation (cv) error rates as a function of the number of LVs of PLS-DA models with two classes (Polymer and Controls). **(a)**; models with five classes (PAM, PEG, PVOH, PVP and Controls) **(b)**; models with nine classes (two different molecular weights each of PAM, PEG, PVOH and PVP and Controls).

To find out how many LVs are most suitable for the model types described in the methodology, the optimum numbers of LVs were determined. Fig 6 shows the error rates of the calculated PLS-DA models. The model that contains only two classes (Fig 6a) reaches the lowest error rate of about 0.340 after 4 LVs. For the model that was trained to consider the 4 polymers and the controls as a class, the lowest error is about 0.316 at 14 LVs (Fig 6b). The smallest error rate for the model that was trained to consider the different molecular weights as separate classes is about 0.369 at 18 LVs (Fig 6c).

With increasing classes, more LVs are needed to keep the error rate low. As expected, the error increases with increasing number of classes or finer discrimination criteria, but the model that considers the four individual polymers and a control (Fig 6b) shows a smaller error than the model that only has to discriminate between polymer vs. control (Fig 6a).

#### Calculated responses for individual classes of PLS-DA models.

**Fig 7:**
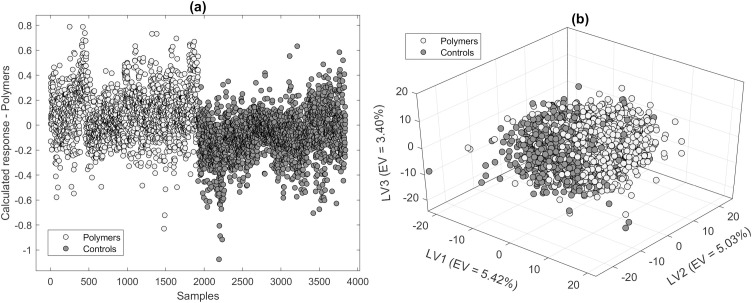
Calculated response for the class “Polymers” of a PLS-DA model (two classes: Polymers, Controls) using 4 LVs (a) and data points of the first three LVs of the same model (b) with explained variance (EV) in %.

To visually assess the model performance in addition to numerical performance parameters, the calculated responses of individual classes of corresponding models can be displayed. Fig 7a shows the calculated responses of the two-class PLS-DA model (polymer vs. control) based on Raman spectra of *A. fischeri*, with the class “Polymer” being selected for visualization.

The large overlap with the control spectra suggests a rather moderate model performance. The plotting of the first 3 LVs (Fig 7b) also shows no clear separation of the data but a clear trend towards successful differentiability. Since the corresponding PLS-DA model only uses a total of 4 LVs, the scatterplot shown (Fig 7b) is very meaningful for model assessment. The representation of the first LVs is less meaningful for models with a high number of LVs, such as the model that considers the four polymers individually (14 LVs). However, by showing the calculated response, it can be made particularly clear which classes can be differentiated well.

**Fig 8:**
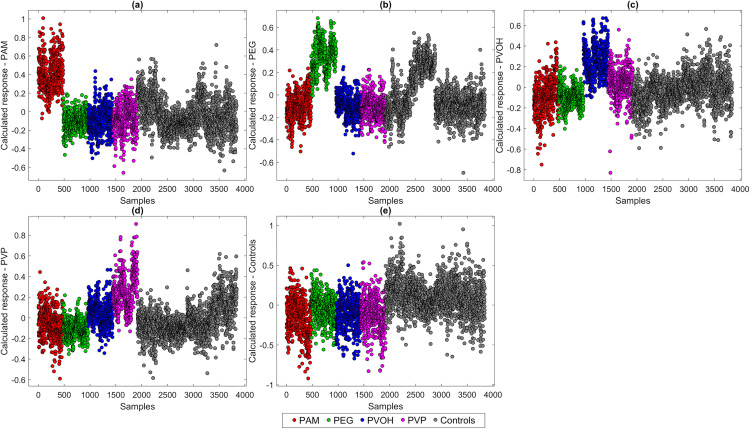
Calculated responses for the classes. PAM **(a)**, PEG **(b)**, PVOH **(c)**, PVP **(d)** and Controls **(e)** of a PLS-DA model (five classes) using 14 LVs.

Fig 8 displays the calculated PLS-DA response values for selected target classes of the five-class model. All sample spectra are included in the plot and are color-coded according to their actual polymer treatment. This allows the comparison of class-specific model responses across all polymer exposures. With PAM incubation, a clear separation of the data can be seen (Fig 8a), which indicates an influence during incubation. Although calculated PEG responses also separate well from the majority of the data, it can be seen that some of the control spectra overlap strongly with the spectra (Fig 8b). As the data shown are systematically arranged, it is immediately apparent that these are the controls that were analyzed in parallel with the PEG sample preparations. This suggests a deviation in the initial cultures of these test series and illustrates the significance of generally possible variance in the biochemical cell state of bacteria despite standardized conditions. PVOH (Fig 8c) and PVP preparations (Fig 8d) can be separated better overall than PEG preparations, but worse than PAM and also in the case of PVP the corresponding parallel controls are more similar than completely independent controls (Fig 8d). Less than half of the calculated responses of the controls themselves show clear separation. This highlights the difficulties of the model to correctly classify the controls and confirms the initial indication of an overall low influence of the polymers during incubation of *A. fischeri*.

**Fig 9:**
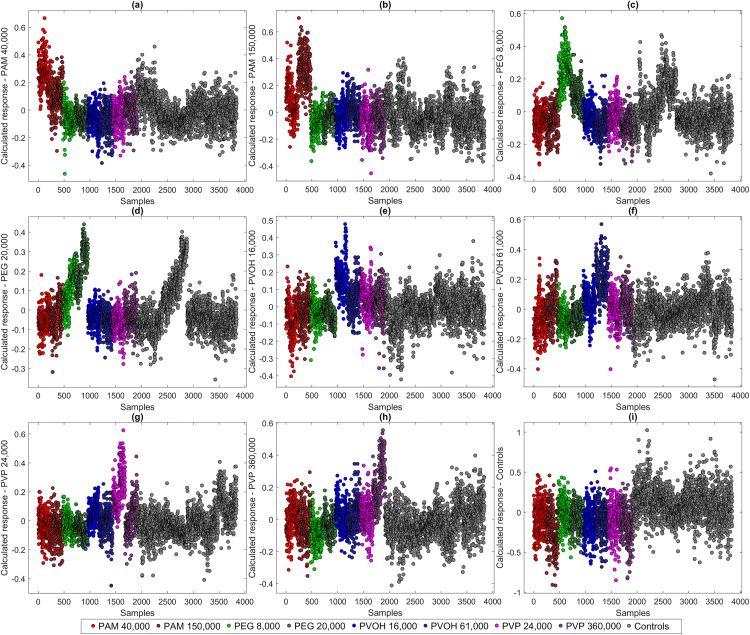
Calculated responses for the classes. PAM 40,000 g/mol **(a)** and 150,000 g/mol **(b)**, PEG 8,000 g/mol **(c)** and 20,000 g/mol, PVOH 16,000 g/mol **(e)** and 61,000 g/mol, PVP 24,000 g/mol **(g)** and 360,000 g/mol **(h)** and Controls **(i)** of a PLS-DA model (nine classes) using 18 LVs.

A model with 18 LVs was also trained which takes into account the individual molecular weights of the four polymers, thus containing nine classes (eight polymers, one control). The calculated responses of these data are shown in Fig 9. Both PAM 40,000 g/mol (Fig 9a) and PAM 150,000 g/mol (Fig 9 b) can be separated relatively cleanly from the rest of the data, but show an overlap with at least half of the respective data. PEG 8,000 g/mol (Fig 9c) and PEG 20,000 g/mol (Fig 9d) can be distinguished quite efficiently from each other, but there is a large overlap with the controls at both molecular weights. Interesting are also peculiarities at PEG 20,000 g/mol (Fig 9d) where even two subclusters can be recognized which are due to the individual two batches. This highlights the issue of general batch-to-batch variations and emphasizes the importance of comprehensive data sets that cover natural or methodical variations. The calculated responses of the PVOH preparations with 16,000 g/mol (Fig 9e) show a large overlap with PVOH with 61,000 g/mol and both PVP preparations. PVOH with 61,000 g/mol (Fig 9f) is separated more clearly overall, suggesting a possible greater influence of this polymer on the Raman spectra of *A. fischeri*. Both PVP 24,000 g/mol (Fig 9g) and PVP with 360,000 g/mol (Fig 9h) show a clear separation with only slight overlapping of other samples. As with the model with only five classes (Fig 8), the calculated responses of the controls show large overlaps with all test approaches. In order to examine the model performance in more detail, other performance parameters are used in addition to the calculated responses.

#### PLS-DA performance parameters.

**Fig 10:**
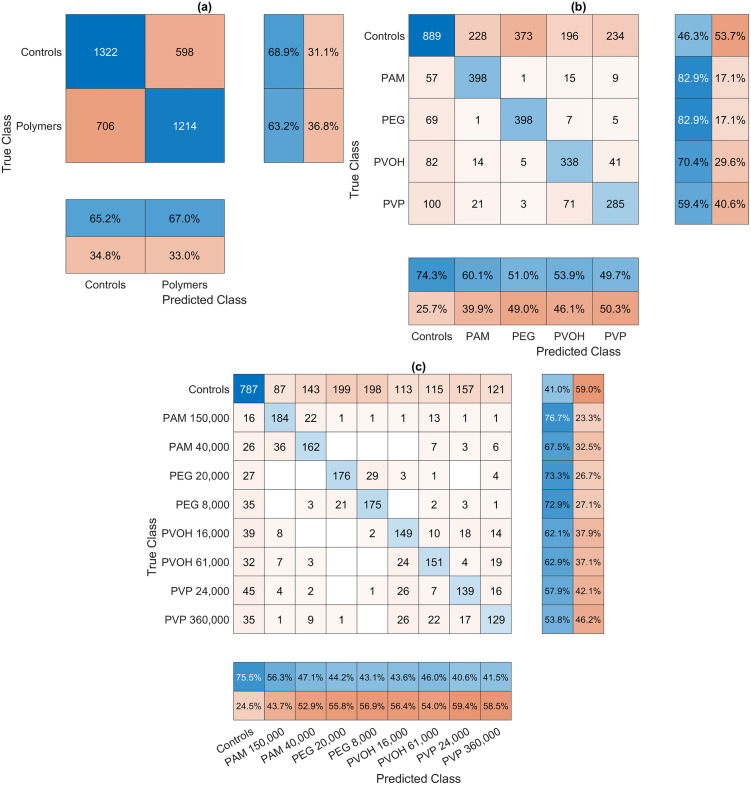
Confusion matrices for the classification of *A. fischeri* Raman spectra after polymer exposure and purification using PLS-DA models of increasing complexity. **(a)** Two-class model distinguishing between polymer-exposed and control bacteria. **(b)** Five-class model differentiating between four polymer types (PAM, PEG, PVOH, PVP) and controls. **(c)** Nine-class model incorporating molecular weight variations within each polymer type. Each matrix displays absolute classification counts, along with row-normalized percentages indicating the proportion of correctly and incorrectly classified samples for each true class, and column-normalized percentages showing the accuracy of each predicted class.

The classification of *A. fischeri* Raman spectra after polymer exposure and purification was evaluated using three PLS-DA models of increasing complexity: a binary model (Fig 10a), a five-class model (Fig 10b), and a nine-class model incorporating molecular weight variants (Fig 10c). In the binary model, classification accuracy was moderate, with 68.9% of polymer-exposed and 63.2% of control spectra correctly classified. Misclassification rates were substantial (31.1% and 36.8%, respectively), and 34.8% of predicted polymer-exposed samples were false positives. These results suggest that Raman spectral changes induced by polymer exposure are still detectable after purification and likely reflect persistent cellular effects rather than residual polymer signals.

The five-class model showed improved discrimination for PAM and PEG (82.9% accuracy each), while PVOH (70.4%) and PVP (59.4%) were less reliably identified. Misclassifications occurred most frequently between PVOH and PVP, suggesting shared spectroscopic features.

Control samples were correctly classified in only 46.3% of cases in the five-class model, meaning that more than half of the control spectra were misclassified as polymer-exposed. The high misclassification rate of control samples suggests that the spectral features distinguishing polymer-exposed bacteria are not fully exclusive and may partially overlap with natural spectral variability in untreated controls. This overlap complicates the separation between exposed and non-exposed samples and indicates that while polymer exposure induces detectable changes, they are not always uniquely identifiable against the background of biological variability.

In the nine-class model, classification accuracy declined, especially for molecular weight variants of the same polymer. PAM 40,000 and 150,000 g/mol showed some differentiation (67.5% and 76.7%, respectively), while PEG 8,000 vs. 20,000 and PVOH 16,000 vs. 61,000 exhibited considerable overlap. PVP variants performed worst (56.3% and 53.9% correct), indicating weak molecular weight-dependent differentiation. This confirms that molar mass influences spectral patterns but does not always allow clear separation. The increased misclassification between molecular weight variants further supports that polymer-induced changes in *A. fischeri* are subtle and not necessarily distinct across molar masses.

Across all models, PAM and PEG exposure induced the most distinct Raman shifts, while PVOH and PVP led to overlapping profiles. The consistent misclassification of control samples, even in the binary model, highlights the limited exclusivity of polymer-induced spectral signatures and highlights the challenge of distinguishing polymer-exposed from unexposed bacterial cells, given the intrinsic spectral variability present even in untreated microbial populations.

#### PLS-DA coefficients.

**Fig 11:**
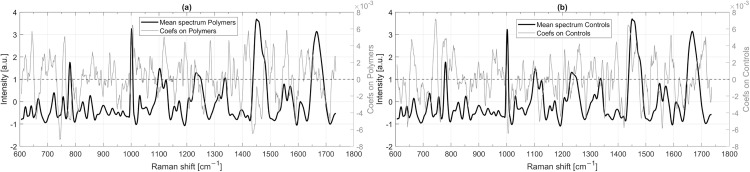
Mean Raman spectrum of samples with polymer incubation and PLS-DA coefficients (Coefs) for this class (Polymers) from the PLS-DA model (two classes) (a) and the same for Raman spectra without polymer contact (Controls) (b).

The above-described results have shown that a pure classification of Raman spectra from *A. fischeri* with polymer exposure is possible in many cases, especially in the five-class model, distinguishing the polymer types and not the molecular weights. In the following, the PLS-DA coefficients of specific classes were examined in order to draw conclusions about Raman spectroscopic changes due to polymer exposure. The mean Raman spectrum of *A. fischeri* exposed to polymers and the corresponding PLS-DA coefficients of the two-class model for this class are shown in Fig 11a. The equivalent data for the control samples, i.e., their mean spectrum and the PLS-DA coefficients for the class “Control”, are presented in Fig 11b. The analysis of PLS-DA coefficients in the two-class model highlights statistically relevant spectral regions that contribute to class separation, offering a promising basis for further biochemical interpretation and hypothesis generation. By identifying the most prominent coefficient values, one can determine which Raman spectral shifts contribute most to distinguishing these two classes. However, due to the moderate classification performance in some models, interpretations should be regarded as preliminary and exploratory in nature. The spectral contributions identified here may indicate potential biochemical differences but require cautious interpretation in the absence of direct biochemical validation. One of the striking PLS-DA coefficients is the spectral region around 745 cm ⁻ ¹, where the PLS-DA coefficients exhibit strong negative values for polymer-exposed bacteria and strong positive values for controls. This indicates that a lower intensity at these Raman shifts increases the likelihood of a spectrum being classified as polymer-exposed, whereas a higher intensity is characteristic of controls. However, no significant signatures can be observed in this area (Fig 11); instead, this coefficient lies exactly between the two signatures at around 723 and 755 cm ⁻ ¹, which are triggered by the ring breathing modes of adenine (723 cm ⁻ ¹) [59] and the symmetric stretching vibration of the indole ring in tryptophan (755 cm ⁻ ¹) [60,61]. Other striking coefficients can be observed at 1438 cm ⁻ ¹ and 1470 cm ⁻ ¹, where polymer-exposed bacteria exhibit lower intensities (Fig 11a) compared to controls (Fig 11b). These two regions flank the well-defined peak at around 1451 cm ⁻ ¹ (Fig 11b), which is commonly associated with CH_2_ scissoring vibrations and is characteristic of both lipids and proteins [61,62]. This might point to changes in membrane-associated regions, potentially involving lipids or structural proteins; however, due to the broad biochemical assignment of CH_2_ bending vibrations, it remains uncertain whether these variations reflect specific molecular interactions or general physiological shifts in *A. fischeri*. Nevertheless, such spectral differences may hint at polymer-induced stress responses or changes in membrane properties, which could include alterations in fluidity or surface-associated interactions.

Another spectral feature that strongly differentiates the two bacterial classes appears around 1004 cm ⁻ ¹. Here, the PLS-DA coefficients show opposite behavior: higher intensities at this Raman shift are associated with polymer-exposed bacteria, while lower intensities indicate control samples. However, the actual peak corresponding to the ring-breathing vibrations of phenylalanine [63,64] appears consistently between 1001 and 1003 cm ⁻ ¹ in both polymer-exposed *A. fischeri* and control samples. The coefficient at 1004 cm ⁻ ¹ likely reflects the decreasing slope of this peak rather than a true peak shift. The PLS-DA model may assign significance to a small but systematic difference in how the peak intensity diminishes toward 1004 cm ⁻ ¹. Since the actual position of the peak caused by phenylalanine remains unchanged, this effect is more likely due to subtle variations in spectral intensity distribution rather than a physical shift. Similarly, 1118 cm ⁻ ¹ and 1559 cm ⁻ ¹, stand out as important indicators of polymer exposure, as their coefficients for polymer-exposed bacteria are among the strongest in the dataset. It is striking that the coefficients (1118 cm ⁻ ¹ and 1559 cm ⁻ ¹) again form exactly between two signatures, namely 1102 and 1125 cm ⁻ ¹ and 1550 and 1572 cm ⁻ ¹ respectively. Therefore, in these cases it is not the distinct bands but the spaces between them that are of great importance for classification.

For control samples, the most striking positive PLS-DA coefficients are found at 1590 cm ⁻ ¹ and 1714 cm ⁻ ¹, meaning that a higher intensity at these shifts makes a spectrum more likely to be classified as a control. Both regions again show no prominent bands and in the case of 1714 cm ⁻ ¹ can even be attributed to a flank at the end of the spectrum which can be more strongly influenced by baseline filters [65].

**Fig 12:**
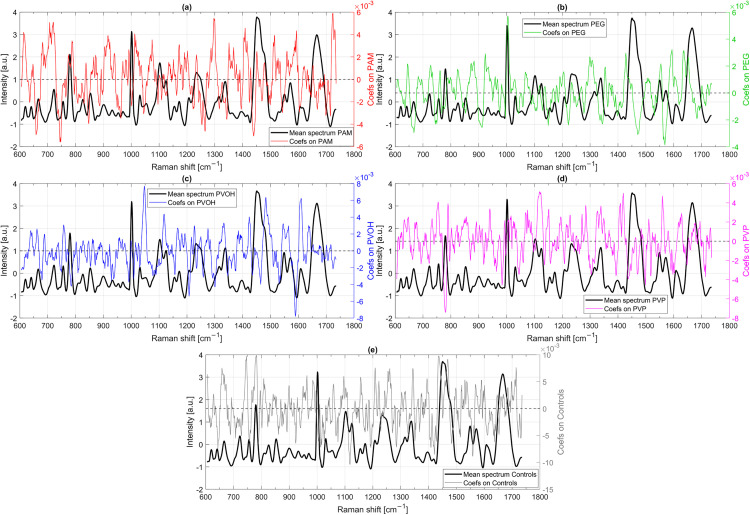
Mean Raman spectrum of samples with individual polymer incubation and PLS-DA coefficients (Coefs) for the classes PAM (a), PEG (b), PVOH (c) and PVP (d) from the PLS-DA model (five classes) and the same for Raman spectra without polymer contact (Controls) (e).

The analysis of PLS-DA coefficients in the five-class model provides insights into which spectral features contribute most significantly to the classification of *A. fischeri* after polymer exposure. Fig 12 presents the mean Raman spectrum of bacteria incubated with each polymer type, along with the corresponding PLS-DA coefficients for PAM (a), PEG (b), PVOH (c), and PVP (d), as well as the coefficients for control samples (e). By examining the most pronounced coefficient extrema, it is possible to determine which Raman shifts serve as key indicators of differentiation.

For PAM-exposed bacteria (Fig 12a), some of the most significant spectral contributions are observed at 616 cm ⁻ ¹ and 719 cm ⁻ ¹, where strong positive coefficients indicate that an increase in intensity at these shifts is characteristic of PAM exposure. Conversely, a negative coefficient at 658 cm ⁻ ¹ suggests that lower intensity in this region is indicative of PAM-exposed spectra. Another strong negative coefficient appears at 746 cm ⁻ ¹, reinforcing that spectra with reduced intensity at this shift are more likely to belong to PAM-exposed bacteria. Additional distinguishing spectral features include 1297 cm ⁻ ¹ and 1440 cm ⁻ ¹, where opposite coefficient values suggest that PAM exposure leads to an increase in intensity at 1297 cm ⁻ ¹ while suppressing signals at 1440 cm ⁻ ¹. A particularly interesting feature is observed at 1724 cm ⁻ ¹, where a strong positive coefficient suggests that this shift is frequently associated with PAM exposure. However, since no major Raman bands typically appear in this region, this feature may be influenced by baseline correction artifacts, which can vary in intensity across different spectra. Despite this, the systematic presence of this shift across samples suggests that it plays a role in classification.

For PEG-exposed bacteria (Fig 12b), a striking spectral feature appears at 1004 cm ⁻ ¹, similar like in the two-class model, where a strong positive coefficient suggests that enhanced intensity in this region is a defining characteristic of PEG exposure. Further positive contributions are seen at 1594 cm ⁻ ¹, 923 cm ⁻ ¹, 643 cm ⁻ ¹, and 1428 cm ⁻ ¹, suggesting that bands in this regions are more pronounced in PEG-exposed spectra compared to other classes. In contrast, negative coefficients at 1568 cm ⁻ ¹, 1470 cm ⁻ ¹, 911 cm ⁻ ¹, and 667 cm ⁻ ¹ indicate that PEG exposure results in reduced Raman intensity at these shifts, reinforcing that spectral suppression in these regions plays an important role in differentiating PEG-exposed bacteria from other classes.

For PVOH-exposed bacteria (Fig 12c), the most dominant spectral feature is observed at 1047 cm ⁻ ¹, where an extremely strong positive coefficient suggests that this shift is indicative of PVOH exposure. The Raman spectra (Fig 5 and Fig 12) do not suggest strong bands in these regions, but the mean value spectra are indeed slightly elevated there, especially for PVOH with 61,000 g/mol. The signature at 1047 cm ⁻ ¹ has already been noticed by Tewes *et al.* [12] is due to polysaccharides [66] and could be related to an increased concentration of carbohydrates due to cell wall changes [12]. Additional positive coefficients appear at 1483 cm ⁻ ¹, 1610 cm ⁻ ¹, and 1118 cm ⁻ ¹, suggesting that these bands are enhanced in PVOH-exposed spectra. In contrast, strong negative coefficients at 1589 cm ⁻ ¹, 1207 cm ⁻ ¹, and 784 cm ⁻ ¹ indicate that lower intensity at these shifts is characteristic of PVOH-exposed bacteria.

For PVP-exposed bacteria (Fig 12d), the most prominent spectral feature appears at about 780 cm ⁻ ¹, where a very strong negative coefficient (similar like PVOH at approximately 784 cm ⁻ ¹) suggests that suppression at this shift is a defining feature of PVP exposure. The 780 cm ⁻ ¹ Raman peak is primarily associated with ring-breathing modes of cytosine and thymine, the pyrimidine bases in DNA and also includes contributions from symmetric phosphodiester stretching vibrations of the DNA backbone [63,66,67]. Further negative coefficients for PVP are observed at 983 cm ⁻ ¹, 1393 cm ⁻ ¹, and 1498 cm ⁻ ¹, indicating that intensity reduction at these bands plays a role in classification. In contrast, positive coefficients at 1118 cm ⁻ ¹, 1559 cm ⁻ ¹, 1408 cm ⁻ ¹, and 1419 cm ⁻ ¹ suggest that these spectral features are more pronounced in PVP-exposed spectra, providing a clear spectroscopic signature for this polymer interaction. Notably, 1408 cm ⁻ ¹ and 1419 cm ⁻ ¹ are two sharply separated peaks, rather than a broad band, indicating distinct spectral contributions in this region.

For control bacteria (Fig 12e), the most pronounced spectral feature appears at about 782 cm ⁻ ¹, where a very strong positive coefficient suggests that increased intensities at this shift is a defining characteristic of controls. Like described above about this region (780 cm ⁻ ¹) is primarily associated with DNA components and the coefficients for PVOH and PVP at this point are strongly negative. The strong positive coefficient at 782 cm ⁻ ¹ for control bacteria, alongside the negative coefficients for PVOH- and PVP-exposed bacteria, suggests that polymer exposure may influence DNA-associated spectral features. The observed differences could indicate changes in DNA conformation, accessibility, or overall cellular state.

Further positive coefficients at 658 cm ⁻ ¹, 746 cm ⁻ ¹, 1437 cm ⁻ ¹, 1469 cm ⁻ ¹, and 1343 cm ⁻ ¹ indicate that these bands are more strongly expressed in control spectra compared to polymer-exposed bacteria. In contrast, negative coefficients at 1012 cm ⁻ ¹, 1118 cm ⁻ ¹, and 1558 cm ⁻ ¹ suggest that control samples exhibit lower intensities at these Raman shifts.

## Discussion

### Luminescence measurements

Since the measured dissolved oxygen content in PVOH is not higher than in other polymer solutions, an elevated oxygen level is unlikely to be the cause for increased luminescence. However, it cannot be completely excluded, as dissolved oxygen represents only one aspect of oxygen availability. Additional factors such as diffusion rates, gradients within the medium, and cellular oxygen consumption also influence the effective concentration experienced by the bacteria [68–70]. Aleskerova *et al.* [71] found a similar effect, as when they performed the immobilization of proteobacteria in a cryogel of PVOH, they found that the immobilization led to an increased intensity and stability of bioluminescence depending on selected species [71]. The increased luminescence in PVOH-exposed *A. fischeri*, particularly at 61,000 g/mol, may result from a combination of protective effects, oxygen retention, and enhanced bacterial viability. PVOH is known for its biocompatibility and stabilizing properties, which can create a microenvironment that supports bacterial metabolism and reduces enzymatic degradation or oxidative stress, thereby helping to preserve luminescence activity [72]. Unlike PEG, PVOH is generally considered biocompatible and does not exhibit strong osmotic effects in many applications [73]. Additionally, its gas barrier properties may help retain oxygen in solution, ensuring a more stable supply rather than increasing diffusion [74]. Moreover, short-term physiological responses, such as enzyme stabilization and improved metabolic efficiency, have been observed in bacteria exposed to PVOH, which could further contribute to sustained or increased luminescence [75]. While the exact mechanism remains unclear in our study, these factors likely contribute to a more favorable physiological state, allowing *A. fischeri* to sustain and even increase its luminescence.

The process described in the introduction for emitting light suggests that a disturbance of the enzyme machinery at various levels could impair light production. However, the assumption that PEG interferes with enzymes or co-factors, for example by binding to active sites or altering enzyme conformation, seems unlikely. The PEG sample with the lowest molar mass showed almost no effect on luminescence, despite smaller molecules generally having higher mobility and surface interaction potential [11]. An alternative explanation is that PEG, particularly at higher concentrations, may exert osmotic stress on cells by creating osmotic gradients across the membrane [76]. This can lead to water loss, cellular dehydration, and physiological stress [77]. This osmotic stress could have a detrimental effect on the metabolic activity and bioluminescence production of *A. fischeri*. Disruption of cell membranes by PEG molecules, which affect the integrity of cell membranes through mechanisms such as membrane permeabilization [78,79], pore formation or disruption of the lipid bilayer [80], cannot be excluded. Further studies using complementary techniques, such as electron microscopy, could help to clarify whether membrane disruption contributes to the observed effects [81]. However, significant membrane disruption appears unlikely under the tested conditions, as bacterial growth was observed in all polymer exposure groups, with comparable optical densities prior to Raman measurements (S2 File). This suggests that the cells maintained structural integrity, allowing for proliferation, and argues against strong lytic or cytotoxic effects. For PEG, it is particularly noticeable that at 8,000 g/mol there is consistently very high luminescence and from 20,000 g/mol there is a drastic decrease in luminescence. However, the trend of decreasing luminescence with increasing molar mass is not observed for PEG between 20.000 g/mol and 35.000 g/mol. For example, the luminescence at 35,000 g/mol is slightly above the 20,000 g/mol sample. Interestingly, there are two different manufacturers for these PEG samples (Table 1), which might support the theory that common impurities or batch variations could also play a role in the luminescence tests with *A. fischeri*. If the inhibitory effect is based on the difference in the larger molar mass, this circumstance seems counterintuitive since it is precisely shorter molecules that are expected to interact more strongly with the microorganisms and high molecular weights are very likely to hinder any uptake of these polymers by biological membranes [11]. However, larger polymer molecules can have more pronounced steric hindrance effects [37], i.e., they can physically hinder the movement or interactions of molecules involved in the bioluminescence process. Polymers with higher molecular weight also increase the viscosity of the solution [82,83]. Higher viscosity can reduce the diffusion rates of oxygen, nutrients, and signaling molecules, potentially slowing the delivery of components essential for the bioluminescence reaction and thereby lowering light output. However, this mechanism alone does not account for the observed effects, as the strongest luminescence reduction occurred with PEG, which exhibits much lower viscosity compared to high molecular weight PAM (e.g., 150,000 or 15,000,000 g/mol) (S7 File). Therefore, viscosity might contribute to the observed luminescence patterns, together with other factors, but is unlikely to be the sole determining cause. Several factors must therefore be involved. The effect that the luminescence decreases with increasing molar mass is relatively clear for PVP. For PVP with 24,000 g/mol and 40,000 g/mol, there are hardly any relative differences in luminescence compared to the positive control, but there is a noticeable decrease in luminescence for the 360,000 g/mol sample (Fig 3). A recent study by Hisar and Oehlmann (2023) [55] reported no adverse effects of WSPs on the luminescence of *A. fischeri*, testing PEG and PAM, among other polymers, at lower molecular weights than those used in the present study. This highlights the added value of our work, as we observed luminescence suppression primarily at higher molecular weights, suggesting a potential threshold effect related to polymer size or aggregation behavior. In addition to the above-mentioned factors influencing a possible decrease in luminescence, it is important to note that commercially purchased polymers do not have to be completely pure. Thus, small residues of possible impurities cannot be entirely ruled out as a cause of luminescence decline in our test setup. Higher molecular weight polymers exhibit higher sorption activity [11,84], which could mean that they exhibit an increased tendency to adsorb potentially harmful substances in solution [85]. This increased sorption activity may have toxicological relevance, as it could alter the transport and fate of contaminants in solution upon contact with biological systems. A purification of the test substances via dialysis, for instance, could provide more clarity in this respect.

A disturbance of the luminescence measurements due to extreme pH values is unlikely. Although some polymer stock solutions initially showed pH values outside the recommended range (6.0–8.5 [56]), the buffering capacity of the culture medium substantially moderated these values during the actual test. Notably, PVP samples initially exhibited the lowest pH values (e.g., 3.82 for 2.5% PVP at 24,000 g/mol), yet showed moderate luminescence after mixing with the medium (pH 6.67). In contrast, PEG samples yielded the lowest luminescence despite displaying pH values within or close to the ideal range. Similarly, the PVOH samples, although slightly acidic prior to buffering, consistently exhibited high luminescence. These observations indicate that luminescence variations cannot be attributed to pH alone, and that other polymer-specific factors must be involved.

### Raman microspectroscopy

#### Spectral features and class separability.

The largely overlapping mean spectra (Fig 5a) confirm that Raman-based classification in this context relies on subtle and distributed spectral features, rather than on sharp biomarker peaks. The elevated variability at ~1550 cm ⁻ ¹ in both PEG and control samples highlights the potential impact of biological batch effects, suggesting that not all deviations are polymer-specific. Likewise, the increased spread in PAM-exposed spectra around 826–853 cm ⁻ ¹ may reflect transient or surface-localized interactions, even if the mean intensity remains unchanged. The PCA (Fig 5b) supports this interpretation, as some group-specific tendencies become visible when multivariate variance is considered. The contribution of specific spectral regions, revealed by the loading vectors (Fig 5c), further corroborates the assumption that polymer exposure, although subtle, can cause class-distinguishing variations in the chemical or structural composition of *A. fischeri* detectable via Raman spectroscopy.

While PCA provides an unsupervised view of spectral variance, supervised methods like PLS-DA enable more targeted discrimination by identifying LVs that best explain differences between predefined classes. These LVs summarize the spectral directions that maximize covariance between spectral patterns and class membership [58]. Their utility becomes evident when examining classification performance across different models: notably, the model discriminating between the four individual polymers and a control (five classes total) yielded a lower cross-validation error than the simpler binary model. This finding suggests that the effects of polymer exposure are not uniform but polymer-specific, pointing toward distinct biochemical or structural alterations in response to each compound. This raises the question of which spectral features contribute most to class discrimination. Analysis of the PLS-DA coefficients provides further insight into these differentiating patterns across the tested models.

Analysis of the PLS-DA coefficients revealed that polymer exposure systematically affects the Raman spectra of *A. fischeri*, with specific spectral variations depending on the polymer type. Particularly in the five-class model, polymer-exposed and control bacteria can be well distinguished, with some significant coefficients occurring between rather than directly on Raman bands. This indicates that polymer exposure not only changes the presence of peaks but also their intensity distributions. Lipid- and protein-associated features in the 1438–1470 cm ⁻ ¹ range show strongly negative coefficients in polymer-exposed bacteria, which could indicate membrane changes or protein modifications. The DNA-associated band at 782 cm ⁻ ¹, pronounced in control bacteria, exhibits lower intensity in polymer-exposed cells, especially in PVOH- and PVP-exposed bacteria. This could indicate changes in DNA accessibility or conformation. In vitro and cellular studies have shown that water-soluble polymers such as PEG can affect DNA conformation and structural dynamics by promoting compaction, relaxation, or destabilization effects, depending on polymer properties and environmental context [86–89]. The strong coefficient at 1047 cm ⁻ ¹ for PVOH-exposed bacteria, despite the absence of a dominant Raman band, suggests an increased concentration of carbohydrate-associated components, possibly due to cell wall modifications. These results demonstrate that polymer exposure not only causes isolated spectral shifts, but also alters broader spectral regions, suggesting systematic biochemical and structural adaptations. While the exact mechanisms remain unclear, the changes in membrane lipids, proteins, and nucleic acids suggest direct polymer-cell interactions or adaptive metabolic responses. The polymer-specific differences in spectral effects, especially the greater similarity between PVOH- and PVP-exposed bacteria, may reflect shared structural or biochemical features. Interestingly, this apparent similarity does not directly correspond to their distinct effects on luminescence, suggesting that Raman-detectable alterations and acute physiological responses may capture different aspects of polymer interaction. To further support spectral interpretation, S8 File compares PLS-DA coefficients with the corresponding class-wise difference spectra for the 5-class model. Interestingly, both representations show a high degree of spectral overlap, suggesting that the observed class contributions are not merely statistical constructs but reflect genuine spectral differences between polymer-exposed and control samples. This alignment reinforces the robustness of the peak assignments. Nevertheless, considering the moderate classification accuracy of some models, further in-depth inspection of the difference spectra may offer additional biochemical insights. While detailed interpretation of difference spectra was not the primary methodological route of this study, the observed overlap with model-derived coefficients highlights their potential as complementary tools. S8 File may thus serve as a methodological extension point for future studies focusing more explicitly on spectral dissection. Future studies should integrate Raman spectroscopy with complementary biochemical and functional assays to clarify whether observed spectral changes result from direct polymer interactions or longer-term cellular adaptations.

A critical factor in Raman-based bacterial classification is the potential influence of variations in the biological starting material, e.g., differences in the selection of bacterial material on agar plates. Different locations on the agar plates could already show bacteria with slightly different physiological status. While the standardization steps used in this study greatly minimize such effects, some biological variation between samples remains to be assumed. However, the fact that systematic spectral differences occur between polymer-exposed and control bacteria indicates that these changes are not only due to possible sample variability, but are most likely related to the polymer interaction itself. If culture-related effects were the predominant factor, one would expect random spectral variability rather than polymer-specific trends, which was not observed. This highlights both the validity and challenges of using Raman microspectroscopy for the exploratory study of bacteria-polymer interactions and highlights the need for careful experimental design of comprehensive and representative data sets and possible complementary biochemical approaches in future studies.

#### Possible role of cell envelope interactions in spectral differentiation.

Among the various interaction modes between WSPs and microorganisms, surface adsorption onto the cell envelope constitutes a primary and broadly acknowledged mechanism. There is consistent evidence that polymer interaction typically begins with physicochemical adhesion to outer cellular structures, regardless of whether a biological effect follows [11,90]. This initial adsorption is largely governed by electrostatic forces, hydrophobic interactions, polymer conformational flexibility, and environmental conditions such as ionic strength and pH [11,90].

The bacterial cell surface presents a complex and chemically heterogeneous interface composed of peptidoglycan, lipopolysaccharides, teichoic acids, proteins, and extracellular polymeric substances (EPS) [91]. Most WSPs, though structurally diverse, possess functional groups capable of engaging in weak but measurable interactions with these components [92–94]. Although the WSPs investigated in this study are non-ionic, it is worth noting that electrostatic interactions represent a well-established mode of polymer-bacteria interaction; for example, cationic polymers, in particular, are well documented to bind electrostatically to anionic constituents of the bacterial envelope, including lipopolysaccharides (LPS) in Gram-negative species and teichoic acids in Gram-positive organisms [95]. In contrast, anionic polymers generally exhibit little to no affinity for LPS under physiological conditions due to the predominantly negative surface charge of the outer membrane [95]. While these charge-based interactions do not apply to the polymers tested here, other weak and reversible mechanisms, such as hydrogen bonding, hydrophobic contacts, or sterically driven adhesion, have been reported to mediate surface-level interactions between WSPs and microbial cells [11,96].

The polymers analyzed in this study (PAM, PEG, PVP, PVOH) are not intentionally designed to target microbial surfaces, but their physicochemical properties could enable such interactions. Review data presented by Duis et al. [11] underscore that WSPs can adsorb to biological surfaces via non-specific interactions, especially in the case of high-molecular-weight, water-soluble species such as PAM and PVP. Surface association is reported to influence cellular aggregation, nutrient uptake, and local microenvironments, effects that may manifest without actual penetration of the cytoplasmic membrane. This is consistent with a broader body of literature on antimicrobial polymers, where surface binding is shown to be the universal prerequisite for subsequent membrane disturbance, even when no direct toxicity is involved [90,97].

Importantly, the reviewed literature converges on the notion that membrane penetration is not a general feature of WSPs unless specific amphiphilic or reactive functionalities are present. Hisar and Oehlmann [55] demonstrated that for a range of commonly used WSPs, including PAM, PEG, and PVOH, no signs of membrane disruption or cytotoxicity were detected in *A. fischeri* bioluminescence assays. This further supports the hypothesis that interactions remain confined to the outer envelope or EPS matrix, rather than extending into the cytoplasmic interior.

From a Raman spectroscopic perspective, surface-localized polymer interactions are particularly relevant, as they may alter the chemical composition or structural organization of outer cell layers in ways that give rise to measurable spectral shifts. For instance, C-O-C stretching vibrations, characteristic of polysaccharide backbones and ether linkages, typically occur in the 950–1150 cm ⁻ ¹ region [63,98]. Likewise, membrane-associated proteins may exhibit changes in the amide I (~1650 cm ⁻ ¹) and amide III (~1250 cm ⁻ ¹) bands, which reflect protein secondary structure and local environment [63,98]. Such alterations could serve as indirect spectral signatures of polymer-cell envelope interaction and have been observed in polymer-exposed microbial systems [12]. In line with this, bacteria exposed to WSPs showed class-specific differentiation using chemometric models, despite the absence of sharp spectral markers [12]. These findings were further supported in our study, where bulk Raman spectra of *A. fischeri* following WSP exposure exhibited reproducible spectral shifts in key fingerprint regions. Nonetheless, such effects should not be over-interpreted due to the only moderate prediction quality on average (Fig 10). Notably, the best classification performance was achieved for PAM, with 82.9% correct cross-validation in a five-class PLS-DA model including all four polymers and a control. In the more granular model incorporating different molecular weight variants, PAM-exposed cells (150,000 g/mol) were still correctly classified in 76% of cases.

These results do not confirm a specific biochemical binding mechanism but suggest that PAM-induced surface effects are particularly pronounced or consistent under the studied conditions. The slightly stronger differentiation observed for PAM may stem from its partially hydrolyzed structure (introducing charged carboxylate groups), optimized charge distribution, and molecular flexibility, all of which have been shown to enhance surface adsorption compared to non-hydrolyzed analogs [99–101]. However, given the exploratory nature of this study, such interpretations must be considered preliminary. In summary, adsorption of WSPs to the bacterial cell envelope emerges as a plausible and well-supported interaction mechanism that aligns with current ecotoxicological, microbiological, and material-scientific evidence. While such surface-level processes may not elicit acute toxicity, they are capable of inducing physicochemical changes that can affect cellular behavior and may be detected through sensitive techniques such as Raman microspectroscopy. Although the rigorous washing protocol employed prior to Raman analysis, comprising three centrifugation and resuspension steps in 20 g/L NaCl solution, ensures substantial removal of unbound polymers, the possibility of trace amounts of weakly adsorbed residues on the bacterial surface cannot be entirely ruled out. However, we emphasize that the Raman spectra did not exhibit characteristic polymer-specific signals, and the observed spectral differences remained subtle and class-dependent. This strongly suggests that the measured signals primarily originate from biological structures rather than residual polymer contamination.

## Conclusion

This study investigated the interactions between WSPs and *A. fischeri* by combining luminescence assays and Raman microspectroscopy, enabling the detection of both acute physiological effects and more persistent biochemical changes. Luminescence measurements revealed polymer- and molar mass-dependent effects: PAM, PEG, and PVP suppressed bioluminescence at higher concentrations and molecular weights, whereas PVOH, particularly at 61,000 g/mol, enhanced luminescence, suggesting a stabilizing or protective effect.

Raman spectral differences between exposed and control bacteria remained detectable even after rigorous purification, indicating that polymer exposure induced cellular alterations extending beyond residual surface contamination. However, physiological heterogeneity within bacterial populations and differences in polymer purity or manufacturing may have contributed to the observed patterns, especially in luminescence outcomes.

The data suggests that WSPs can elicit subtle, polymer-specific effects at the bacterial cell envelope, which are detectable via sensitive vibrational spectroscopic methods. These effects likely reflect physicochemical surface interactions rather than membrane penetration or acute toxicity. While the combined approach of luminescence and Raman analysis provides valuable insights, further research integrating biochemical assays, such as enzyme stability, membrane integrity, and oxygen uptake, will be essential to clarify the underlying mechanisms and distinguish direct polymer effects from secondary physiological responses.

While Raman microspectroscopy is an exceptionally sensitive technique, its full analytical power in detecting subtle polymer-bacteria interactions emerges less from exploratory use alone and more through supervised modeling. To enhance mechanistic insight, future studies should aim to correlate Raman-based findings with independent reference methods.

## Supporting information

S1 File Luminescence measurements with additional highly soluble PVOH sample. (PDF)

S2 File OD_600_ readings of samples for Raman microscopic investigation. (PDF)

S3 File Exemplary selection of four measurement areas of a sample. (PDF)

S4 File Complete raw luminescence spectra of all negative control samples. (XLSX)

S5 File Dissolved oxygen contents of the highest concentrated (2.5% (w/v)) polymer samples for luminescence measurements. (PDF)

S6 File pH values of all polymer solutions before mixing with the buffer medium. (PDF)

S7 File Exemplary comparative viscometric analysis of PEG 35,000 g/mol and PAM 15,000,000 g/mol. (PDF)

S8 File Difference spectrum (mean spectrum of samples with individual polymer incubation minus mean spectrum of controls) and PLS-DA coefficients (Coefs) for the classes PAM (a), PEG (b), PVOH (c) and PVP (d) from the PLS-DA model (five classes). (PDF)
