# Supplementary material for: Exploring interactions of Aliivibrio fischeri with water-soluble polymers using bioluminescence and Raman microspectroscopy
Source: PLoS One. 2025 Sep 16;20(9):e0330775. doi: 10.1371/journal.pone.0330775 (PMC12440198; doi:10.1371/journal.pone.0330775)
Supplement: S1 File — (PDF) [file pone.0330775.s001.pdf]

Supplementary Material S1: Luminescence measurements with additional highly soluble PVOH sample.

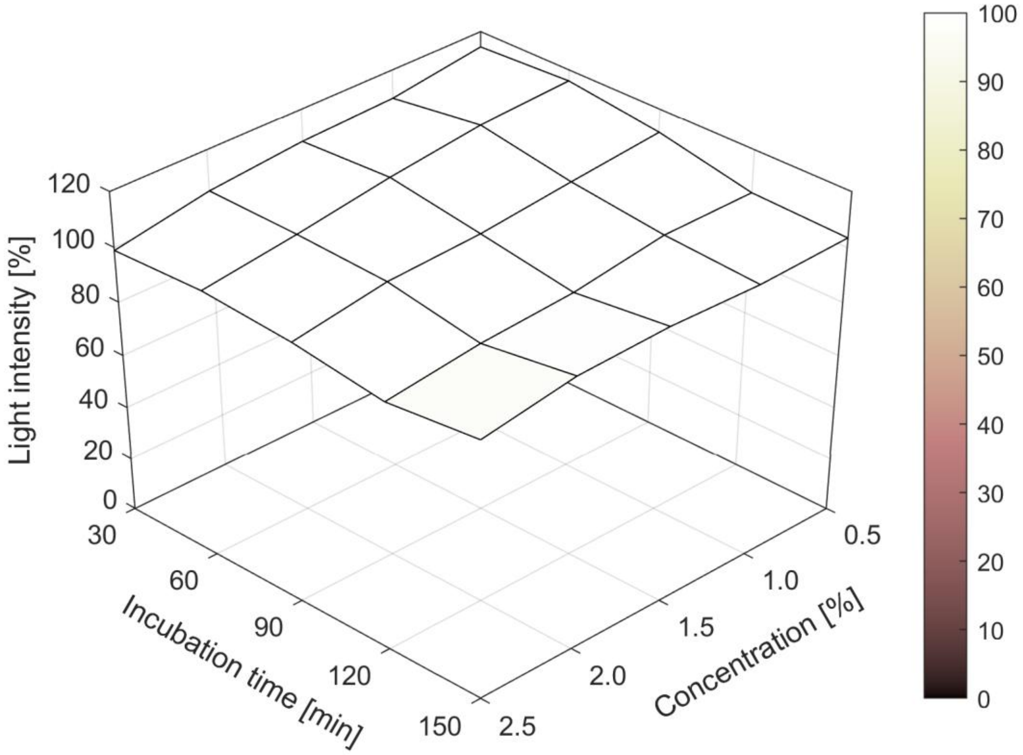

Concentration [% (w/v)]	Incubation time [min]				
	30	60	90	120	150
	Light intensity [%]				
**0.5**	114.53	116.03	111.71	104.55	103.45
**1.0**	109.70	114.31	108.49	104.49	102.36
**1.5**	108.40	110.10	104.97	99.55	103.54
**2.0**	105.21	104.75	103.41	97.48	102.51
**2.5**	98.92	100.18	97.92	93.36	97.08

Polyvinylalkohol (PVOH) (Waldeck GmbH & Co KG, Münster, Germany) with molar mass not provided by the manufacturer but measured with same method described in Tewes T.J., Kaba A., Schacher F.H., Bockmühl D.P. (2025) A Combined Raman Spectroscopy and Chemometrics Study of the Interaction of Water-Soluble Polymers with Microorganisms. Spectrosc. J. 3. https://doi.org/10.3390/spectroscj3010007 with 339,000 g/mol. The size exclusion chromatography (SEC) method used, in particular the calibration based on polymer standards, leads to a systematic overestimation of the molar mass. Based on a power fit, a realistic molar mass of approx. **67,678 g/mol** is obtained for the analyzed sample.
