# Supplementary material for: Exploring interactions of Aliivibrio fischeri with water-soluble polymers using bioluminescence and Raman microspectroscopy
Source: PLoS One. 2025 Sep 16;20(9):e0330775. doi: 10.1371/journal.pone.0330775 (PMC12440198; doi:10.1371/journal.pone.0330775)
Supplement: S2 File — (PDF) [file pone.0330775.s002.pdf]

Supplementary Material S2: OD_{600} readings of samples for Raman microscopic investigation.

Polymer and molecular weight M_W [g/mol]	Culture	Batch 1		Batch 2	
		OD initial	OD after incubation	OD initial	OD after incubation
PAM 40,000	Starting	0.521	2.591	0.577	2.657
	test	0.708	1.961	0.736	1.785
	control	0.759	2.157	0.763	2.118
PAM 150,000	Starting	0.551	2.567	0.546	2.493
	test	0.694	1.785	0.642	1.770
	control	0.729	2.118	0.680	2.103
PEG 8,000	Starting	0.558	2.457	0.552	2.839
	test	0.716	2.372	0.802	2.393
	control	0.738	2.423	0.844	2.514
PEG 20,000	Starting	0.565	3.007	0.571	2.088
	test	0.838	2.498	0.460	1.607
	control	0.900	2.636	0.508	1.848
PVOH 16,000	Starting	0.536	2.344	0.573	1.898
	test	0.628	2.394	0.458	2.087
	control	0.644	2.450	0.441	2.032
PVOH 61,000	Starting	0.556	1.858	0.521	2.225
	test	0.451	1.956	0.602	2.311
	control	0.437	2.050	0.626	2.342
PVP 24,000	Starting	0.565	2.076	0.556	1.542
	test	0.476	2.025	0.322	1.705
	control	0.504	2.052	0.339	1.779
PVP 360,000	Starting	0.556	2.065	0.560	1.695
	test	0.505	2.142	0.362	1.855
	control	0.514	2.203	0.389	1.869
