# Supplementary figures and images for: Exploring interactions of Aliivibrio fischeri with water-soluble polymers using bioluminescence and Raman microspectroscopy
Source: PLoS One. 2025 Sep 16;20(9):e0330775. doi: 10.1371/journal.pone.0330775 (PMC12440198; doi:10.1371/journal.pone.0330775)

**Supplementary Material S3: Exemplary selection of four measurement areas of a sample.**

**20× lens**

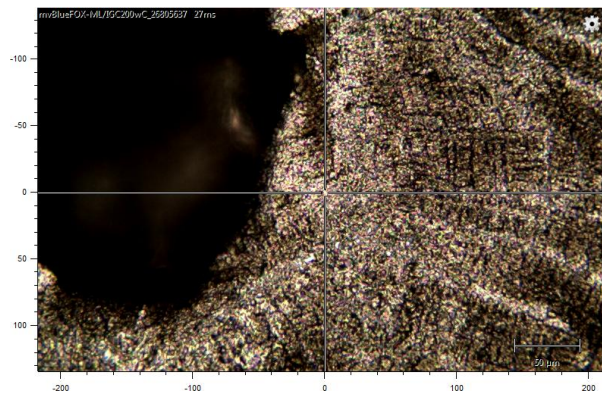

**100× lens**

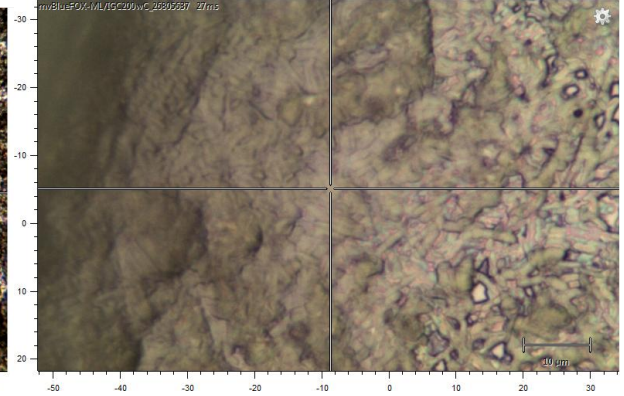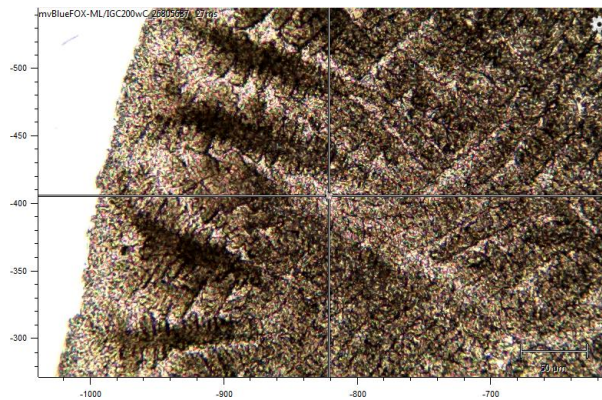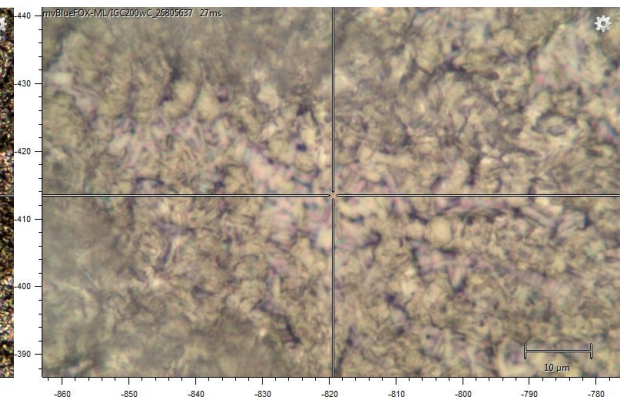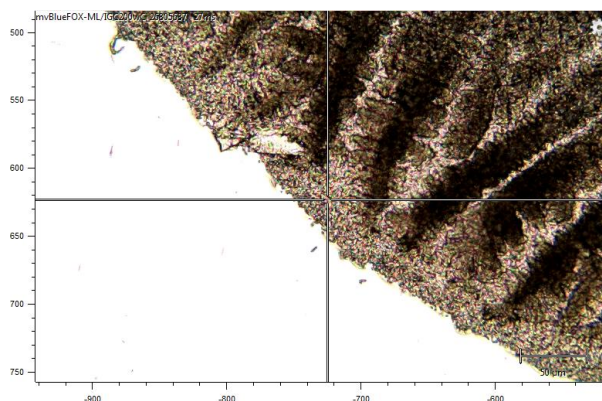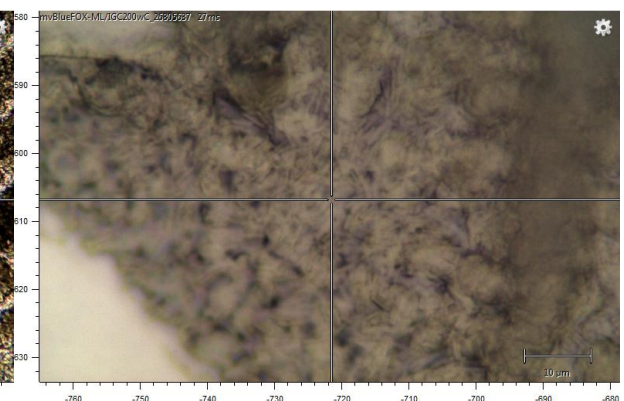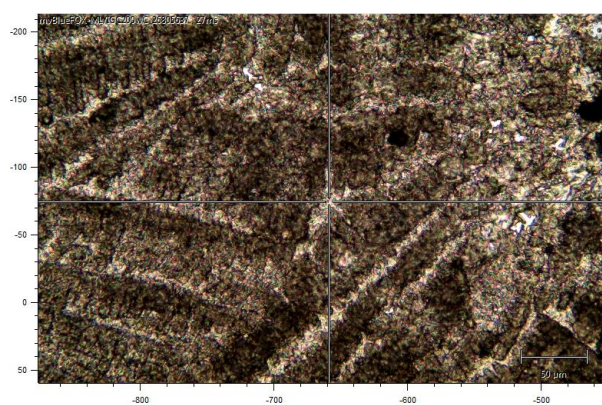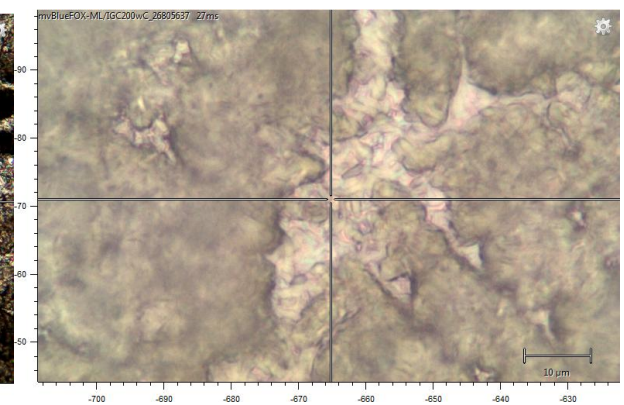

Supplement: S3 File — (PDF) [file pone.0330775.s003.pdf]
