# Supplementary material for: Exploring interactions of Aliivibrio fischeri with water-soluble polymers using bioluminescence and Raman microspectroscopy
Source: PLoS One. 2025 Sep 16;20(9):e0330775. doi: 10.1371/journal.pone.0330775 (PMC12440198; doi:10.1371/journal.pone.0330775)
Supplement: S5 File — (PDF) [file pone.0330775.s005.pdf]

Supplementary Material S5: Dissolved oxygen contents of the highest concentrated (2.5% (w/v)) polymer samples for luminescence measurements.

Polymer	MW [g/mol]	Dissolved oxygen [mg/L]
PAM	40,000	8.5
	150,000	8.3
	15,000,000	7.6
PEG	8,000	8.2
	20,000	8.3
	35,000	8.6
PVOH	16,000	8.6
	47,000	7.6
	61,000	7.9
PVP	24,000	8.8
	40,000	8.6
	360,000	8.4
