# Supplementary material for: Exploring interactions of Aliivibrio fischeri with water-soluble polymers using bioluminescence and Raman microspectroscopy
Source: PLoS One. 2025 Sep 16;20(9):e0330775. doi: 10.1371/journal.pone.0330775 (PMC12440198; doi:10.1371/journal.pone.0330775)
Supplement: S6 File — (PDF) [file pone.0330775.s006.pdf]

Supplementary Material S6: pH values of all polymer solutions before mixing with the buffer medium.

Polymer	Molecular weight [g/mol]	Concentration (w/v)				
		2.5%	2.0%	1.5%	1.0%	0.5%
		pH				
PAM	40,000	8.91	8.20	7.93	8.00	6.79
	150,000	6.00	6.06	5.97	6.56	6.34
	15,000,000	6.48	6.76	6.64	6.62	6.57
PEG	8,000	6.08	5.86	6.01	5.79	5.84
	20,000	6.23	5.68	6.02	5.89	5.87
	35,000	4.95	4.96	5.33	6.53	6.12
PVOH	16,000	5.80	5.80	5.81	5.83	5.91
	47,000	5.85	5.85	5.85	5.85	5.92
	61,000	5.83	5.83	5.83	5.83	5.91
PVP	24,000	3.82	3.87	3.90	3.96	4.08
	40,000	3.90	3.92	3.97	4.03	4.20
	360,000	3.77	3.93	4.07	5.89	5.68
