# Supplementary material for: Exploring interactions of Aliivibrio fischeri with water-soluble polymers using bioluminescence and Raman microspectroscopy
Source: PLoS One. 2025 Sep 16;20(9):e0330775. doi: 10.1371/journal.pone.0330775 (PMC12440198; doi:10.1371/journal.pone.0330775)
Supplement: S7 File — (PDF) [file pone.0330775.s007.pdf]

Supplementary Material S7: Exemplary comparative viscometric analysis of PEG 35,000 g/mol and PAM 15,000,000 g/mol.

Polymer	Molecular weight [g/mol]	Concentration (w/v)	
		0.5%	
		Dynamic viscosity [mPas]	Torque [µNm]
**PAM**	15,000,000	239.9	4.38
**PEG**	35,000	12.00	0.202
**Distilled water**	-	7.998	0.135

The measurements were carried out according to the enclosed manufacturer's recommendations, i.e. 500 ml polymer solution in a 600 ml glass beaker. Spindle “L3” at 30 rpm was used and a measuring time of 5 minutes. Sample temperatures were 21°C.
