# Supplementary material for: Exploring interactions of Aliivibrio fischeri with water-soluble polymers using bioluminescence and Raman microspectroscopy
Source: PLoS One. 2025 Sep 16;20(9):e0330775. doi: 10.1371/journal.pone.0330775 (PMC12440198; doi:10.1371/journal.pone.0330775)
Supplement: S8 File — (PDF) [file pone.0330775.s008.pdf]

Supplementary Material S8: Difference spectrum (mean spectrum of samples with individual polymer incubation minus mean spectrum of controls) and PLS-DA coefficients (Coefs) for the classes PAM (a), PEG (b), PVOH (c) and PVP (d) from the PLS-DA model (five classes).

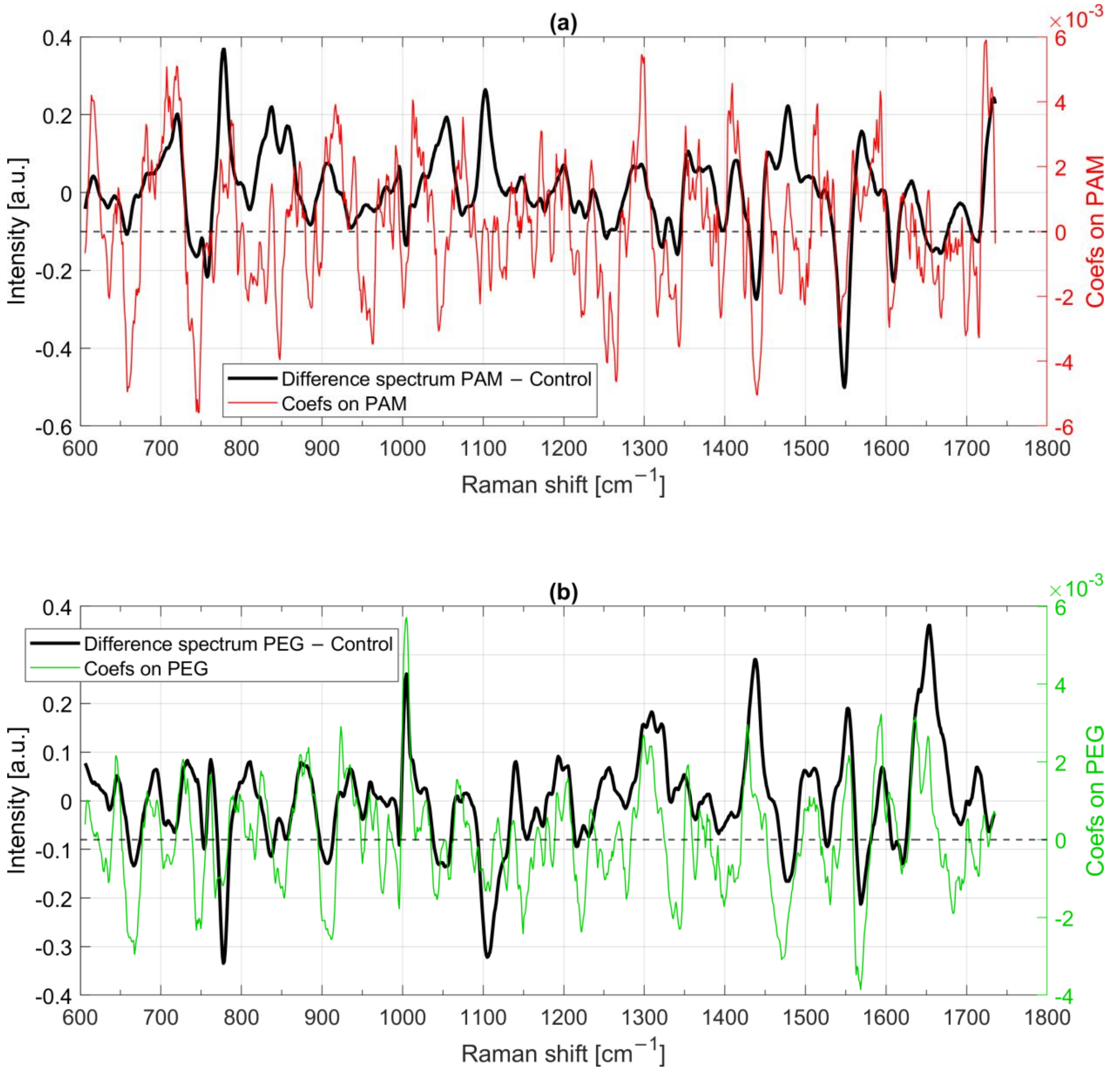

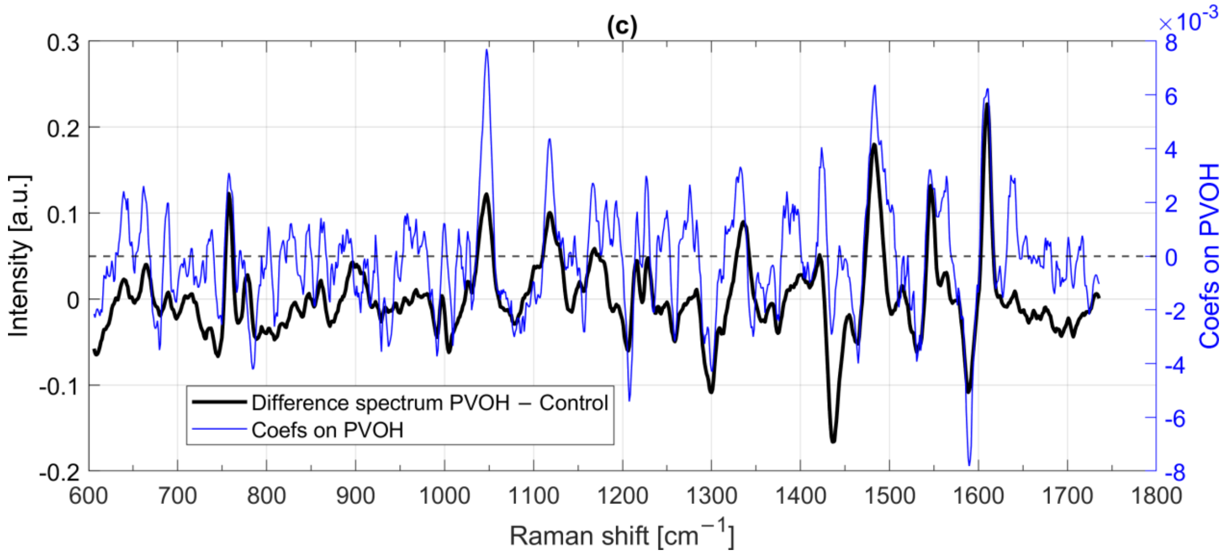
(c)
Intensity [a.u.]
Coefs on PVOH
Raman shift [cm^{-1}]
Difference spectrum PVOH − Control
Coefs on PVOH
×10^{-3}

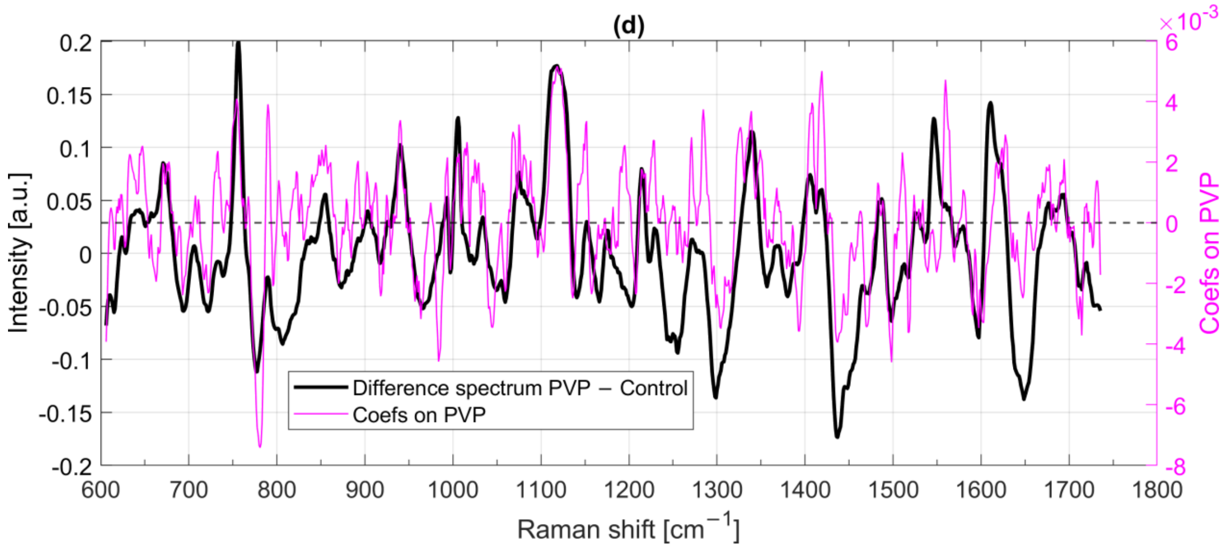
(d)
Intensity [a.u.]
Coefs on PVP
Raman shift [cm^{-1}]
Difference spectrum PVP − Control
Coefs on PVP
×10^{-3}
